# Operational Modal Analysis of Near-Infrared Spectroscopy Measure of 2-Month Exercise Intervention Effects in Sedentary Older Adults with Diabetes and Cognitive Impairment

**DOI:** 10.3390/brainsci13071099

**Published:** 2023-07-20

**Authors:** Fei Zhao, Machiko Tomita, Anirban Dutta

**Affiliations:** 1 Department of Rehabilitation Science, School of Public Health and Health Professions, University at Buffalo, Buffalo, NY 14214, USA; fzhao6@buffalo.edu (F.Z.); machikot@buffalo.edu (M.T.); 2 School of Engineering, University of Lincoln, Lincoln LN67TS, UK

**Keywords:** type 2 diabetes mellitus, functional near-infrared spectroscopy, muscle near-infrared spectroscopy, cognitive impairment, operational modal analysis

## Abstract

The Global Burden of Disease Study (GBD 2019 Diseases and Injuries Collaborators) found that diabetes significantly increases the overall burden of disease, leading to a 24.4% increase in disability-adjusted life years. Persistently high glucose levels in diabetes can cause structural and functional changes in proteins throughout the body, and the accumulation of protein aggregates in the brain that can be associated with the progression of Alzheimer’s Disease (AD). To address this burden in type 2 diabetes mellitus (T2DM), a combined aerobic and resistance exercise program was developed based on the recommendations of the American College of Sports Medicine. The prospectively registered clinical trials (NCT04626453, NCT04812288) involved two groups: an Intervention group of older sedentary adults with T2DM and a Control group of healthy older adults who could be either active or sedentary. The completion rate for the 2-month exercise program was high, with participants completing on an average of 89.14% of the exercise sessions. This indicated that the program was practical, feasible, and well tolerated, even during the COVID-19 pandemic. It was also safe, requiring minimal equipment and no supervision. Our paper presents portable near-infrared spectroscopy (NIRS) based measures that showed muscle oxygen saturation (SmO2), i.e., the balance between oxygen delivery and oxygen consumption in muscle, drop during bilateral heel rise task (BHR) and the 6 min walk task (6MWT) significantly (*p* < 0.05) changed at the post-intervention follow-up from the pre-intervention baseline in the T2DM Intervention group participants. Moreover, post-intervention changes from pre-intervention baseline for the prefrontal activation (both oxyhemoglobin and deoxyhemoglobin) showed statistically significant (*p* < 0.05, q < 0.05) effect at the right superior frontal gyrus, dorsolateral, during the Mini-Cog task. Here, operational modal analysis provided further insights into the 2-month exercise intervention effects on the very-low-frequency oscillations (<0.05 Hz) during the Mini-Cog task that improved post-intervention in the sedentary T2DM Intervention group from their pre-intervention baseline when compared to active healthy Control group. Then, the 6MWT distance significantly (*p* < 0.01) improved in the T2DM Intervention group at post-intervention follow-up from pre-intervention baseline that showed improved aerobic capacity and endurance. Our portable NIRS based measures have practical implications at the point of care for the therapists as they can monitor muscle and brain oxygenation changes during physical and cognitive tests to prescribe personalized physical exercise doses without triggering individual stress response, thereby, enhancing vascular health in T2DM.

## 1. Introduction

The Global Burden of Disease Study (GBD 2019 Diseases and Injuries Collaborators) showed that diabetes significantly increases the burden of disease with a 24.4% increase in age-standardized disability-adjusted life years [1]. Additionally, projections from the Centers for Disease Control and Prevention indicate that the burden of Alzheimer’s Disease Related Dementias in the United States is expected to double by 2060. It is known that persistently elevated glucose levels in diabetes can lead to structural and functional changes in proteins throughout the body, and the spread of protein aggregates in the brain is implicated in the progression of Alzheimer’s Disease (AD) [2]. The term “type 3 diabetes” reflects the fact that AD represents a form of diabetes [3] and physical activity with diet can be important for diabetes management [4]. According to the American Diabetes Association [5], adults with all types of diabetes should participate in more than 150 min of moderate intensity physical activity weekly for at least three days a week. However, previous research has shown that only 25% of older adults with diabetes met this recommendation [6], which can be due to low physical performance threshold, poor cardiorespiratory fitness, and other functional limitations in older adults with type 2 diabetes mellitus (T2DM) [7,8,9]. Older adults with T2DM often lack stamina and feel tired during prolonged single-time exercise, which may lead to a cessation of exercise training and obesity [10,11]. Therefore, older adults with T2DM often become sedentary resulting in a decline in their physical conditions [12] where adjusting the intensity of the regular exercise programme to fit T2DM individual capacity should be investigated to disrupt this vicious cycle. To address this need, a low to moderate intensity individualized exercise programme was developed considering the reduced capacity in sedentary older adults with T2DM [13]; however, the beneficial effects on the brain and muscle needed to be established. In this paper, we present the development of the near-infrared spectroscopy (NIRS)-based measures (some we published in conferences [14,15,16]) to investigate the 2-month exercise intervention effects in sedentary older adults with diabetes and cognitive impairment. We investigated 2-month exercise intervention effects on the low-frequency oscillations (LFO) (0.05–0.2 Hz) and very-low-frequency oscillations (VLFO) (<0.05 Hz) [16,17] based on our preliminary findings [16] using power spectral density analysis that revealed a significantly lower relative power in the 0.021–0.052 Hz (smooth muscle autonomic innervation) frequency band in elderly subjects with T2DM during the Mini-Cog three-item recall test. Here, we hypothesized that the data-driven modal analysis based on our related works on systems modeling [18,19,20] will be able to disentangle the 2-month exercise intervention effects leveraging the effects on the dynamic properties of neurovascular coupling system in the frequency domain including endothelial metabolic [16] related VLFO (<0.02 Hz), neurogenic sympathetic related VLFO (>0.02 Hz and <0.05 Hz), and the neurovascular and myogenic influenced LFO (0.05–0.2 Hz) [16,17]. Notably, in our preliminary study [16], we found a drop in the oscillatory power in the 0.01–0.02 Hz frequency band during the Mini-Cog three-item recall test that was more pronounced in the elderly subjects with T2DM.

We developed a 2-month exercise intervention based on several published studies that demonstrated that frequent but short single training sessions at moderate intensity can promote insulin sensitivity and reduce fatigue in older adults with T2DM [21]. Moreover, researchers have developed an individualized duration for a single exercise session for young adults and then planned multiple sets of that exercise session based on the changes in the muscle oxygenation during physical tasks and recovery stages [22,23]. Here, the superiority of multiple moderate intensity exercise sets compared with a single high intensity set may be appropriate exercise programme for older adults too. Since older adults with T2DM lack endurance and fatigue quickly [24], exercising at shorter duration may fit into their capacity better which may lead to better adherence. Then, on the cardiovascular side, resistance training has been associated with acute reductions in the elastic properties [25,26] while aerobic exercise training increases the elastic properties of central arteries [27]. Since acute reductions in the elastic properties [25] are sustained for <60 min after the completion of resistance exercise, it is likely due to the acute crosstalk between the autonomic nervous system and blood vessels [28]. Importantly, a reduction in arterial compliance has been shown after 2 months of resistance training where no further changes were observed between the 2nd and the 4th month of the exercise intervention [29]. Here, we postulate that any exercise intervention related change in the elastic properties of cerebral blood vessels should affect the dynamic properties of the neurovascular coupling system in the frequency domain based on computation modeling [19] and experimental evidence on the modulation of contractile states of the vascular smooth muscle cells [30]. Then, vulnerability to neurovascular coupling (NVC) impairments (AD-like) has been indicated with a decreased NVC for the default mode network while the dorsal and salience-ventral attention networks had an increased NVC that may be compensatory processes [31] linking T2DM and AD [32]. Such compensatory brain processes may be subserved by neurometabolic alterations [32] where hyperglycemia may lead to glutamate-induced excitotoxicity. In glutamate-induced excitotoxicity, dysfunctional NVC can be related to the elevated astrocytic [Ca^2+^] due to neuronal hyperactivation that suppresses arteriolar [Ca^2+^] oscillations [33] which inhibits [Ca^2+^] oscillations in parenchymal microvessels in the very low frequency (VLFO) (<0.05 Hz). Then, the blood−brain barrier glucose transport may also be adapted to the bioenergy needs during neuronal hyperactivation [34]. Therefore, exercise intervention related effects on the neurometabolic coupling [35] in T2DM and the role of lactate in brain energy metabolism during neural activation [36] are crucial where bioenergetic failure may share neurovascular unit (including blood−brain barrier) dysfunction with molecular and biochemical features [3] in type 3 diabetes mellitus [32]. Indeed, exercise can provide a powerful stimulus for mitochondrial function [37] where mitochondrial pyruvate metabolism may play a key role [38], e.g., slow stretching exercises with muscle contractions [39].

In the current study, we aimed to study task-related muscle and brain oxygenation changes to 2-month moderate intensity exercise intervention in sedentary older adults with diabetes and cognitive impairment that may help determine exercise effectiveness. The cerebral oxyhemoglobin (oxyHb) and deoxyhemoglobin (deoxyHb) changes to Mini-Cog tasks (accessed on 12 June 2023 https://www.alz.org/media/documents/mini-cog.pdf) [40] and muscle oxygen saturation (SmO2) changes to biped tasks were measured with the NIRS technique [41]. Published studies [42,43,44] have demonstrated the adaptation of skeletal muscles to acute exercise and exercise training that can ameliorate metabolic dysfunction and prevent chronic disease [45]. Here, the metabolism in slow-twitch oxidative skeletal muscle [46,47] may be the key for understanding the response to moderate-intensity physical activity in T2DM [12] in terms of dose response [48] measured with muscle NIRS [49]. The muscle NIRS method has developed rapidly in the last 20 years for measuring changes in hemoglobin and myoglobin oxygenation level in blood and skeletal muscle [49]. Muscle NIRS method estimates how oxygen delivery meets oxygen utilization during exercise [49] by delivering near-infrared (NIR) light at wavelengths ranging from 650 to 900 nm into the muscle tissue and measures the amount of scattered light to estimate the muscle tissue properties. Different levels of SmO2 cause varied absorption rates in muscle tissues where the concentrations of two states of hemoglobin plus myoglobin, bound to oxygen and unbound to oxygen, in the tissue can be measured [50,51,52]. Muscle oxygen saturation, known as SmO2, is calculated as the percentage of oxygen bound in the total hemoglobin plus myoglobin concentration. In recent years, muscle NIRS has been used in clinical studies among people with chronic diseases, including patients with muscle myopathies [53], peripheral arterial disease [54,55], muscle atrophy in multiple sclerosis patients [56], heart failure [57,58], by measuring the match between oxygen delivery and utilization. Some studies utilizing muscle NIRS techniques analyzed tissue oxygenation changes after an incremental power test to find the exercise threshold [43,59,60,61]. For example, a study used the difference between oxy hemoglobin plus myoglobin and deoxy hemoglobin plus myoglobin to detect the time point at which the athletes reached the plateau at the physiological level [60]. Moreover, our previous study [13] exhibited a significant and positive correlation between the oxidative capacity expressed by changes in SmO2 and physical performance, including balance, gait speed, and endurance, during a one-time low intensity heel raise physical stimulus among older adults. We developed a normalized SmO2 change rate (NSmO2 rate) [13], that is, a ratio between the rate of SmO2 drop to the amount of exercise, expressed by a SmO2 drop slope (numerator) and the duration of exercise (denominator). The slope of SmO2, defined by the difference between maximum SmO2 and minimum SmO2 divided by the time to reach minimum SmO2, was calculated by linear regression that corresponded with local perfusion [61]. Older adults with higher local perfusion [61] can have a better match between oxygen delivery and oxygen utilization, and they showed an increased level of physical performance. However, the relationship between disease pathology and vascular problems with respect to SmO2 and cerebral oxyHb among patients with diabetes is unclear [61,62]. Here, endothelial vascular function and the capacity to increase cardiac output during exercise can be impaired in T2DM [62]. Moreover, impaired vasodilation [63,64], especially homogenous blood flow distribution within the microvasculature, can be affected by the mechanical properties of the endothelial glycocalyx [65]. Here, endothelial glycocalyx can be a shield against diabetic vascular complications [66] that can be negatively affected by the exercise induced acute stress responses especially at high-intensity interval exercise-induced oxidative stress [67]. Then, maximal exercise capacity was found to be positively associated with the microvascular glycocalyx thickness at baseline [68] and a deteriorated glycocalyx could initiate cardiovascular disease pathology [69] which can be a concern in high-intensity one-size-fits-all resistance training [25,26]. Therefore, individualized dosing of precision exercises may be key when shedding of glycocalyx components is a ubiquitous process that can change the physiology of the endothelium during dose response [70]. Then, animal study has shown a reduction of the glycocalyx length with diabetes progression that correlated with an increasing level of glycated hemoglobin and decreased endothelial nitric oxide (NO) production [71]. Decreased endothelial NO production can affect vascular tone [72] and vasomotion [73]—an emergent phenomena [74]—where optimal exercise can facilitate laminar shear for enhanced endothelial function [75]. Moreover, physiological performance can be mediated, at least in part, by decreased bioavailability of NO that may be ameliorated with regular aerobic exercise [76] thereby improving endothelial reactivity by reducing myoendothelial calcium signal spreading [77]. Therefore, progressive resistance training and moderate intensity walking were proposed for the 2-month exercise intervention where moderate intensity aimed at endothelial glycocalyx protection [78,79].

In this study on the development of NIRS measures, it was postulated that 2-month moderate intensity exercise intervention effects on the endothelial NO production [71] (due to mechanotransduction at the endothelial glycocalyx [65]) can affect vascular rhythmic activity [73], even in the absence of neural activity, especially in the small vessels (<0.3 mm) [16] enhanced by the nonlinear rheology of blood [80]. Then, a reduced myoendothelial calcium signal spreading will also affect the oscillations in the parenchymal microvessels in the very low frequency (<0.05 Hz) [33]. In our prior work [16], we found that hemodynamic oscillations, measured with functional near-infrared spectroscopy (fNIRS), had a lower relative power in the 0.01–0.052 Hz frequency band in the elderly subjects with T2DM when compared to age-matched controls during a Mini-Cog three-item recall test. The potential reasons can be impaired small vessel function due to dysfunctional cytosolic oscillator, membrane oscillator, metabolic oscillator [81], vasoactive signaling [82], and smooth muscle autonomic innervation (0.021–0.052 Hz). Then, the biomechanical properties of the endothelial glycocalyx [83] may determine the Fahræus−Lindqvist-driven oscillations in the small vessels (<0.3 mm) [16] that are due to the non-linear rheological properties of blood [84] flowing through the microvascular networks [85]. In this study, we applied prefrontal fNIRS covering the nodes of the default mode network and the dorsal and salience-ventral attention networks [31] that can measure the global oscillatory behavior of the local microvascular network. Then, the postulated oscillatory dysfunction was investigated [16] using operational modal analysis [86] of the cerebral flow-induced vibrations measured with fNIRS. Published work shows that the pulsatility of large blood vessels shows stiffening in T2DM that is then unable to cushion the pulsatile blood flow driven by cardiac cycle [87]. However, parenchymal microvessel oscillations in the very low frequency (<0.05 Hz) [33] may represent interactions between endothelial NO production [71], Fahræus−Lindqvist-driven flow, which may be crucial for microvascular health in hyperglycemia [88]. Moreover, endothelial glycocalyx promotes homogenous blood flow distribution within the microvasculature [65] that is crucial for the homogeneous diffusion of metabolites in the neurovascular tissue [16]. In the current study, changes in the prefrontal oxyhemoglobin (oxyHb) and deoxyhemoglobin (deoxyHb) concentration during Mini-Cog from resting levels were used that was motivated by the prior work on fNIRS during memory encoding and retrieval in healthy [89]. Jahani et al. [89] showed activation of the left dorsolateral prefrontal cortex during memory encoding while memory recalling resulted in activation in dorsolateral prefrontal cortex bilaterally. In the current study, we aimed to examine the effect of 2-month moderate-intensity aerobic and resistance exercise training on the muscular oxygen saturation (SmO2) changes to physical activity, prefrontal oxyHb and deoxyHb changes to cognitive activity, and operational modal analysis (OMA) of the hemodynamic responses to a cognitive task (Mini-Cog). We applied the Covariance-Driven Stochastic Subspace Identification (SSI-COV) method [90] for OMA where unknown neurovascular inputs that evoked the hemodynamic response were considered as realizations of white noise processes. Moreover, we postulated that 2-month exercise intervention in sedentary older adults with T2DM and cognitive impairment can attenuate prefrontal overactivation during Mini-Cog tasks [91]. We selected the Mini-Cog test since it was suitable for rapid evaluation of cognition with a sensitivity of 76% and specificity of 73% [91] which may be improved by combining with portable brain imaging measures [41].

## 2. Methods

### 2.1. Study Design

Our current study on the NIRS measures is based on the prospectively registered clinical trials (accessed on 12 June 2023 https://www.clinicaltrials.gov/ct2/show/NCT04626453, https://www.clinicaltrials.gov/ct2/show/NCT04812288) that are published in the doctoral thesis of the first author, Fei Zhao [92]. The study design was approved by the ethics committee at the University at Buffalo Institutional Review Boards (IRB), USA, with the approval code, STUDY00004297, and approval date: 23 September 2020. Our experimental study on the NIRS measures has two groups: an Intervention group of older sedentary adults with T2DM (N = 20) and a Control group (N = 40) of healthy older adults who can be active or sedentary. Table 1 shows the T2DM intervention group and the two Control groups, active healthy (N = 20) and sedentary healthy (N = 20), who were age and sex matched with the Intervention group. For age matching, the subjects in the control group were within a two-year difference from the matched person in the T2DM Intervention group. Only the T2DM Intervention group received our 2-month exercise intervention and were evaluated at baseline and follow-up. The Control group did not perform our exercise intervention and was evaluated only once. Both baseline and follow-up data from the T2DM Intervention group were compared with each other as well as with the baseline data from the Control group. T2DM Intervention group performed moderate intensity resistance exercise and performed walking faster than usual on every alternate day for six days a week, as described next. Subjects in the T2DM Intervention group were contacted every two weeks by phone for encouragement to continue the exercise programme.

### 2.2. Development of the 2-Month Exercise Intervention

Based on the American College of Sports Medicines (ACSM) [93], participants performed combined aerobic and resistance exercise, which consisted of progressive resistance exercise and walking at a faster speed than leisure walking, i.e., at a moderate intensity, for six days per week. Participants were encouraged to do progressive resistance exercises and walk for about 20 min a day. Progressive resistance exercise was suggested to be performed every other day for three days a week and walking as aerobic exercise was on alternate days for three days a week for about 20 min a day. The subjects decided which 6 days they perform and 1 day they did not perform the exercises. ACSM [93] suggested an exercise set of 8–12 repetitions for 8–10 exercises, which involves major muscle groups and 10–15 repetitions for progression. Here, a “repetition” is a single execution of an exercise, e.g., one heel raise is one rep or “repetition”, and 10 heel raises are 10 reps. Then, a “set” is a collection of reps where the intensity and progression were tailored for Intervention group in our study. We reduced the number of resistance exercises to four that included 1. knee extension flexion with ankle weights for both the legs, 2. flexion with ankle weights for both the legs, 3. Chair stand with the support, and 4. heel raises with the support of a chair or table. Based on a systematic review of progressive exercises [94], these movements were suggested for T2DM patients to lower blood glucose levels. One-minute stretching was recommended as a warm-up before the four movement sets and the participants had on an average 30 s break between the movement sets.

**Figure 1:**
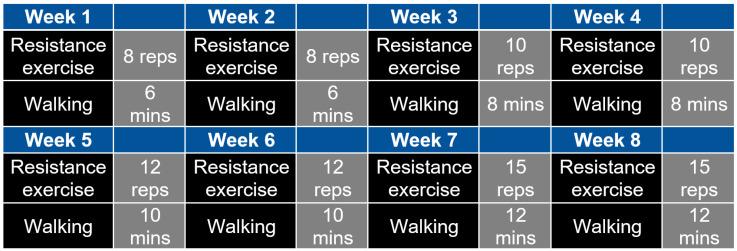
An example of moderate intensity exercise intervention programme.

To determine individualized optimal intensity for exercise at the baseline, we let participants choose a comfortable ankle weight for knee flexion and extension and tested the maximum number of reps for those movements. The maximum number of repetitions was also tested at baseline for the chair stand but without using ankle weights. The participants had a 30 s rest between the movement sets. For the heel raise activity, we obtained the maximum number of heel raises at baseline from the bilateral heel raise test. A marker was placed on the wall above the participants’ heads at the maximum height, and they were encouraged to reach the maximum height each time. The resting time was identified for participants’ muscles to recover back to the baseline SmO2 level after the heel raise task. Then, the resting time between each heel raise movement set was selected at an individual level. Here, each session took approximately 15–20 min. For the exercise programme, the repetitions for each movement set were greater than 50% of the maximum reps at baseline. Figure 1 shows an example where 8 reps in movement sets were applied in weeks 1 and 2 while 10 reps were applied in the weeks 3 and 4. Here, the subject picked in weeks 1 and 2 three movement sets that increased to 4 movement sets for the weeks 3 and 4. From the week 5 onwards, the number of repetitions in each movement set were 60% of the maximum reps at baseline. Then, the subject picked in weeks 5 and 6 three movement sets that increased to four movement sets for the weeks 7 and 8.

For the moderate intensity walking intervention at a brisk pace, first the heart rate reserve (HRR) was calculated using the maximum and resting heart rates. The maximum walking speed was acquired from the 6 min walking test (6MWT) where we measured their heart rate right after the 6MWT. At weeks 1 and 2, participants started at their 60% HRR and then increased to 65%, 70% to 75% of their HRR every two weeks. The total duration for one session of resistance exercise or walking was 6–15 min as shown by an example in Figure 1. We recommended that the participants walk indoors in their dining room or other places with ample space and sufficient light. This was to avoid carpets which can cause them to fall. If the distance in the dining room was too short, we suggested walking between rooms. The duration of the daily exercise containing two sessions (morning and evening) was 152–256 min. In addition to resistance exercise, the weekly walking exercise was 120–180 min. One-time walking or resistance training was considered as one session and the total number of sessions was 96 (2 times per day, 6 days a week, for 8 weeks). Figure 1 presents an example of the moderate intensity exercise intervention programme from the doctoral thesis of the first author, Fei Zhao [92].

### 2.3. Sample Size

**Figure 2:**
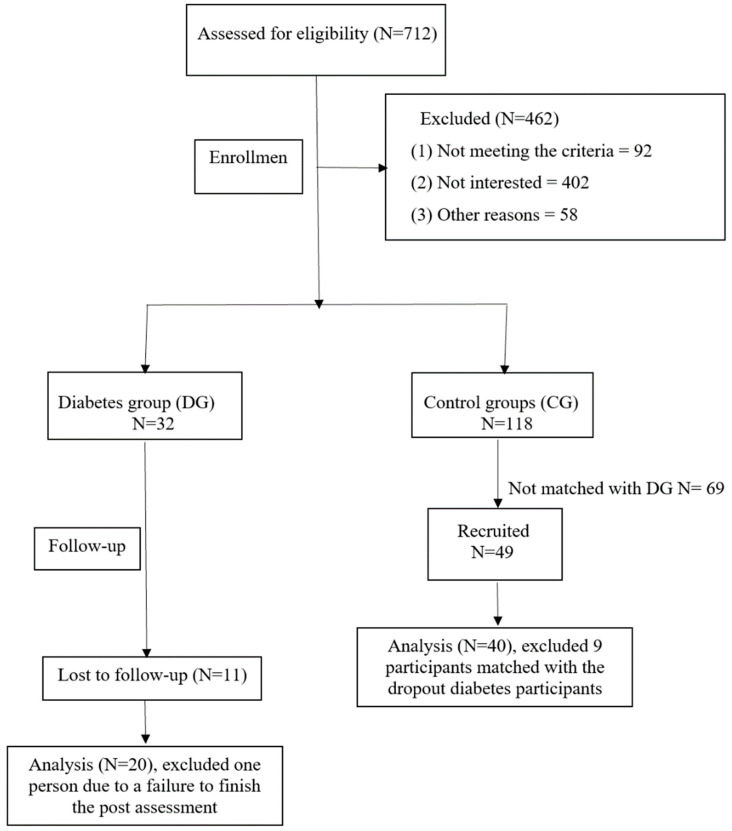
CONSORT flow diagram for screening, eligibility, and included participants.

Sample size was determined by power analysis using the expected medium−large effect size from our preliminary study (f = 0.36) [13] at a significance level to achieve 80% statistical power. The recruitment considered a 20% attrition and screening failure rate in the diabetes group and a 10% screening failure in the control groups—please see the CONSORT flow diagram (accessed on 12 June 2023 https://www.equator-network.org/reporting-guidelines/consort/) in Figure 2. For the Intervention group of older sedentary adults with T2DM, we recruited 32 participants, but 11 participants (34%) dropped out before the follow-up visit due to COVID-19, or other health conditions unrelated to COVID-19, and one did not complete the follow-up assessment. For the Control group, 118 were found eligible, 49 were matched with T2DM Intervention group, and the data from 40 people were matched for the follow-up. A higher dropout rate occurred in our study than expected which was partly due to the COVID-19 conditions. The demographic information is provided in Table 1.

### 2.4. Inclusion and Exclusion Criteria

Figure 2 shows the CONSORT flow diagram (accessed on 12 June 2023 https://www.equator-network.org/reporting-guidelines/consort/) for screening, eligibility, and included participants. The first author, Fei Zhao, performed the screening and included participants under the guidance of Machiko Tomita and Paresh Dandona, who is the University at Buffalo faculty expert on diabetes. For the Intervention group, inclusion criteria were sedentary older adults 60 years or older living in communities with T2DM, ambulatory with or without devices, and able to follow simple exercise instructions without assistance. Additionally, the intervention group must be cleared by their primary physician to perform moderate intensity exercise. The Control group was healthy older adults 60 years or older living in communities who are either sedentary or active and were age and gender matched with the intervention group. The inclusion criteria for the sedentary individuals, either healthy (in the Control group) or T2DM (in the Intervention group), were that they perform weekly moderate intensity exercise that is less than 30 min a day less than five days a week, except for leisurely walking and light housework based on the Rapid Assessment of Physical Activity [95]. Moderate intensity exercises include, for example, walking faster than usual, aerobics class, cycling, swimming, strength training, swimming, and snow shoveling. For the active Control group of healthy older adults 60 years or older living in communities, the additional inclusion criteria were being able to (1) walk six minutes as quickly as possible without any adverse symptoms including extreme pain on feet or joints, dizziness, vertigo, or nausea; and (2) perform moderate-intensity exercise weekly more than 30 min a day five days a week or vigorous physical activities every week more than 20 min a day or three days a week. Vigorous physical activities include stair machines, jogging or running, tennis, or badminton. The exclusion criteria were (1) foot deformities, cuts, blisters or amputation, Achilles tendonitis, joint replacements in the past three months, Parkinson’s disease, retinopathy such as severe glaucoma, current or uncontrolled vestibular disorders; (2) current cardiopulmonary diseases, vascular diseases, or stroke; (3) dementia indicating the inability to follow exercise instructions independently; (4) blood glucose level above 400 mg/dL or HbA1c > 8.0%, (5) type 1 diabetes, and (6) local skin fold larger than 40 mm that will be detrimental to NIRS. Additional exclusion criteria were (1) current smoking or smoking within the last 12 months; (2) currently receiving or planning to receive physical therapy within two months, and (3) lack of English proficiency.

### 2.5. Experimental Test Protocol

The experimental test protocol was approved by the ethics committee at the University at Buffalo Institutional Review Boards (IRB), USA, with the approval code, STUDY00004297, and approval date: 23 September 2020. For the recruitment, a phone screening was conducted based on the inclusion and exclusion criteria once the participants contacted the investigator. After the written consent, assessments were made in the same order for all participants and specific instructions for the exercise program were provided. For the assessments, first, we made an appointment for participants to visit a University at Buffalo clinical laboratory. After the participants arrived, we explained the study using a consent form. After obtaining the written consent of a participant, we asked the person to test the blood sugar level if it was not measured on that day. After checking that blood glucose is less than 400 mg/dL, we measured participants’ blood pressure and heart rate for safety considerations to continue the assessment. HbA1c and body fat were also measured. Subsequently, demographic and health information were obtained. After collecting basic questions, recording devices were attached—first, we placed a fNIRS device on a participant’s forehead to measure cerebral oxyHb. The Mini-Cog was performed three times with three different steps. Each time, there were three Mini-Cog steps: (1) memorizing three words, (2) drawing a clock following given instructions, and (3) recalling three words given in first step. When the tests were completed, the fNIRS device was removed. Participants were asked to drink water if necessary. Then, the muscle NIRS device was placed on the gastrocnemius lateralis. Bilateral heel raise task (BHR) [96] was the first assessment and then rest until SmO2 recovered to the resting level (3 min break). Then, the 6MWT [97] was performed, followed by the rest until SmO2 recovered. Finally, blood pressure, heart rate, fatigue, and blood glucose were measured again to ensure that the participants were doing good. Then, we offered water and food containing glucose. The entire assessment took about 2.5 h per person at each visit for the baseline measures and for the follow-up. After the assessments, the instructions for the 2-month home exercise were explained to the Intervention group and printed materials were provided. In addition, a pedometer was provided to wear while walking. The investigators conducted a home safety assessment to ensure that the home environment was safe for exercise. After each assessment, $50 was given to each Intervention group participant at the baseline as well as at the follow-up visits, while $40 was given to each Control group participant. The Intervention group participants received biweekly reminder phone calls and were asked to fill out an exercise log every time they exercised. We monitored the adherence rate, that is the proportion of prescribed exercises taken. Local data storage allowed direct control over data security measures. We implemented robust security protocols, access controls, and encryption techniques to protect sensitive patient information and ensure compliance with applicable privacy regulations (e.g., Health Insurance Portability and Accountability Act—HIPAA).

#### 2.5.1. Recruitment

Once the research received approval from the IRB, recruitment flyers were sent out to various locations including senior centers, churches, independent living facilities, assisted living facilities, and the University at Buffalo Clinical and Translational Science Institute (Figure 1 shows the CONSORT flow diagram). The institute provided a list of individuals who met the major inclusion criteria. Interested individuals reached out to the investigator directly, while those who did not make contact received either a phone call or a mailed flyer as a follow-up. For the intervention group, a consecutive sampling method was employed. As for the control group, participants were selected from convenience samples, ensuring age and sex matching. The first author, Fei Zhao, led the recruitment under the guidance of Machiko Tomita and Paresh Dandona who is the faculty expert on diabetes.

#### 2.5.2. Pre- and Post-Intervention Measurements

##### Pre-Intervention Cognitive Performance

Table 2 shows the cognitive performance where the Intervention group at baseline was found to have cognitive impairment based on the Mini-Cog and Trail Making test [98] at *p* < 0.01 when compared to the age- and gender-matched participants in the Control group. Here, for the Trail Making test for older adults 60 years or older with ≥16 years of education, 39.55 s for Trail Making test Part A and 93.45 s for Trail Making test Part B for the Intervention group at baseline fall into the Low Average (9th—24th %ile) and Below Average (3rd—9th %ile) percentiles, respectively [98].

##### Cerebral Oxygenation Measures

**Figure 3:**
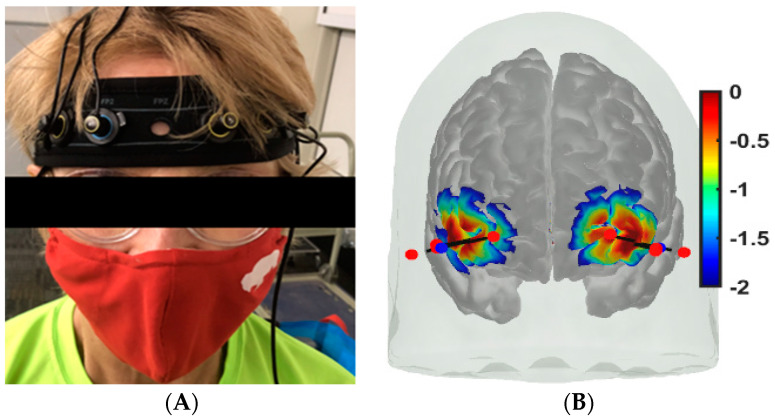
(**A**) Pre- and post-intervention measurements: subject wearing fNIRS headband with two receivers and four transmitters. (**B**) fNIRS headband optode sensitivity profile (default range of 0.01 to 1 or −2 to 0 in log10 units) with NIR light sources in red and the two detectors in blue.

The fNIRS device used to assess cerebral oxygenation [99] was the OctaMon+ (Artinis Medical Systems, Einsteinweg 17, 6662 PW Elst, Elster, The Netherlands), which could measure 8-channels of prefrontal oxyhemoglobin (oxyHb) and deoxyhemoglobin (deoxyHb) at 50 Hz. A headband with two receivers and four high-power transmitters was worn on the forehead for long-separation channels—see Figure 3A. Two low-power transmitters were aligned with the two receivers to measure systemic artifacts in a few healthy control subjects—see Figure 3B. The fNIRS has established good concurrent validity with functional magnetic resonance imaging (fMRI) with support that fMRI and fNIRS have similar vascular sensitivity based on finger tapping tasks in healthy young (r = −0.70, *p* < 0.001) and older adults (r = 0.82, *p* < 0.001) [100,101]. The prefrontal cortex was measured bilaterally, as shown in Figure 3B, during Mini-Cog tasks.

The headband with fNIRS optodes was attached to the forehead (see Figure 3A). In Figure 3B, optode sensitivity values are displayed logarithmically with a default range of 0.01 to 1 or −2 to 0 in log10 units. The optode montage covered the middle frontal gyrus (orbital part) and the superior frontal gyrus (dorsolateral part) bilaterally (see Figure 3B) covering the nodes of the default mode network and the dorsal and salience-ventral attention networks [31]. Block design is one of the most widely used paradigms in fNIRS experiments that was used in our study. In block design, the same test was repeated several times and the average hemodynamic response was calculated. Here, a block design comprises of repeated blocks of tasks (≥10 s) and a rest time of usually 10–20 s. The rest period allowed the task stimulus-evoked hemodynamic response to return to the baseline. The Mini-Cog has three steps repeated three times and a 10 s rest was given between each step. Data pre-processing was performed using HOMER3 toolbox [102] in Matlab (Mathworks Inc., Natick, MA, USA).

**Figure 4:**
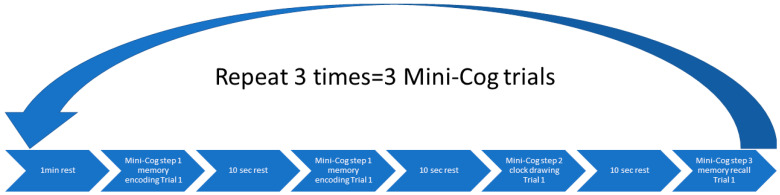
Block design for the cognitive tasks—Mini-Cog.

A series of offline pre-processing steps were performed in HOMER3 software (accessed on 12 June 2023 https://openfnirs.org/software/homer/), as presented next. The processing stream was made up of five functions. The first function is to convert the raw optical intensity signal into optical density (function: hmrR_Intensity2OD). The second function is motion artefact detection and correction to eliminate noises due to scalp movements such as frowning or raising eyebrows. The motion artefact was removed using a hybrid method based on a spline interpolation method and Savitzky–Golay filtering (function: hmrR_MotionCorrectSplineSG). The parameters are *p* of 0.99, FrameSize_sec of 10, and turnon of 1. Then the third function is band-pass filtering (function:hmrR_BandpassFilt:Bandpass_Filter_OpticalDensity). A high-pass filter was set up at the cut-off frequency of 0.01 Hz to remove baseline shifts and low-frequency noise. A low pass filter was set at the cut-off frequency of 0.1 Hz to remove high-frequency noises caused by heartbeat, respiratory rate, and instrument noise. Then, the pre-processing was followed by conversion to oxyHb and deoxyHb concentration with partial path length factor (function: hmrR_OD2Conc) of 1.0. The last is computation of the hemodynamic response function (HRF) where we aimed at investigating the shape of the HRF without imposing canonical modeling constraints [103]. In prior work, Jahani and colleagues [89] used the time range of 25 to 50 s (i.e., less than 60 s) because in this time range the hemodynamic signal reached steady state. We used block average (function: hmrR_BlockAvg: Block_Average_on_Concentration_Data) to determine the hemodynamic response during the stimulation period from the resting state to 60 s after the stimulus was administered (trange = [−5, 60]). This function gave an average HRF of three trials for Mini-Cog—see Figure 4. Moreover, we used AtlasViewer software (accessed on 12 June 2023 https://openfnirs.org/software/homer/) to visualize the cortical activation to facilitate anatomical interpretation of the fNIRS data. Here, cortical activation graphs show the average prefrontal cortex activation using the HRF at a group level and based on the optode sensitivity profile shown in Figure 3B. Since we did not have subject-specific magnetic resonance imaging to create an individual head model, we used the “Colin27” digital brain atlas (accessed on 12 June 2023 http://mcx.space/wiki/index.cgi?action=browse&id=MMC/Colin27AtlasMesh&oldid=MMC/CollinsAtlasMesh) for the reconstruction of brain activation images with the default regularization scaling parameter = 0.01. Finally, we used the NIRS Brain AnalyzIR Toolbox [104] for statistical analysis that provided a finite impulse response (FIR) model which allowed an unconstrained deconvolution and estimation of the full hemodynamic response [103]. We used default pipeline (nirs.modules.default_modules.single_subject with resampling at 0.5 Hz to reduce computational load) on the raw data, including AR-IRLS for correcting motion and serially correlated errors [105], for the statistical analysis using FIR basis function (nirs.design.basis.FIR with binwidth = 1, nbins = 30, isIRF = false)—the statistical analysis is described in further details in the Statistical analysis subsection.

In this study, we introduced OMA of the task stimulus-evoked hemodynamic responses (oxyHb, deoxyHb) pre-processed in the NIRS Brain AnalyzIR Toolbox [104]. The low pass filter was set at the cut-off frequency of 0.2 Hz to capture the LFO (0.05–0.2 Hz) where the cognitive load excitation of the prefrontal cortex was considered as realizations of white noise processes. OMA is for the extraction of modal parameters (natural frequency, damping) where we used a covariance-driven Stochastic Subspace Identification (SSI-Cov) approach that also provided modal parameter uncertainty estimates (details are in the Supplementary Materials). Here, the principal component analysis provided the projection space for the modal solution [106]. We also used frequency domain decomposition [107] to compare with the SSI-Cov results from time domain decomposition. In our prior computational work on experimental modal analysis (EMA) using multi-modal signals including electroencephalogram [18], we have estimated modal parameters by simple peak picking and found modulation by external excitation with transcranial electrical stimulation (tES). The stabilization diagram for modal analysis (‘modalsd’ in Matlab) allowed selection of stable modes (frequency, damping) that do not change substantially with the change in the size of the model (i.e., estimated number of modes). From our prior computational work on EMA of NVC [18,19,103], a maximum of 30 modes were estimated in the current study using OMA. For OMA, we were interested in the physiological modes based on our prior computational work on EMA of NVC [18,19,103] with frequencies less than 0.2 Hz that were found relevant for fNIRS including LFO (0.05–0.2 Hz) and VLFO (<0.05 Hz) [16,17]. Finally, we applied a multi-stage clustering for automated OMA built on the definitions of the pole distance and modal assurance criterion [108] using the Modal Toolkit (accessed on 12 June 2023 https://code.vt.edu/vibes-lab/modal-analysis).

##### Muscle Oxygenation Measures

We used a wearable device, Moxy-1 (Fortiori Design LLC, 1155 West Shore Dr SW, Hutchinson, MN, USA), which measured SmO2 percentage (%) of the gastrocnemius lateralis muscle at 1 Hz sampling frequency. The device was attached by a band across the belly of the calf muscle in the dominant leg of the participants and was further secured with the tape. We first located one third along the line from fibular tuberosity to calcaneus tuberosity of the gastrocnemius muscle and attached the muscle sensor at the junction point. The distance from the strap to the lateral malleolus was recorded in each participant to allow reproducible positioning. The validity of muscle NIRS in the exercising muscles has been established to measure SmO2 at low to moderate exercise intensities [109]. Here [109], SmO2 measured by the Moxy device was in the vastus lateralis during cycling that showed a very high correlation between trials (Spearman’s order rank coefficient: r = 0.842–0.993), and an intraclass correlation was also high (r = 0.773–0.992, *p* < 0.01). SmO2 measured by the Moxy device was also highly correlated with oxygen uptake and heart rate (r = 0.71–0.73, *p* ≤ 0.01) [109].

We measured two muscle oxygenation features, SmO2 drop during a physical task and SmO2 recovery speed after the physical task [13], where the statistical analysis is described in the Statistical analysis subsection. The drop in SmO2 was defined as the difference between the maximum and minimum SmO2 during the physical tasks, the BHR and the 6MWT. Then, SmO2 recovery starts immediately after the participant finishes the physical tasks and sits down. The SmO2 level increases when the recovery period begins. Some participants’ SmO2 reaches the maximum during the recovery period and stays stable after that. To calculate SmO2 recovery speed, the first step was to use the maximum SmO2 during recovery to subtract the SmO2 level at the beginning of the recovery period. Then, the difference in SmO2 is divided by the time taken from the beginning to the maximum (seconds), which is SmO2 recovery rate (SmO2% per s) [13].

##### Cognitive and Physical Function Tasks

The Mini-Cog and Trail Making test [98] examined cognition while the physical performance was measured using the 6MWT and the BHR task. The Mini-Cog instrument assesses short-term memory and visuo-constructive abilities. There are three steps [110], see Figure 4, where the first step is to measure the ability to repeat three words immediately after the experimenter. The second step is to draw a clock based on the verbally assigned time in 3 min. The last step is to recall the three words from the first step. A systematic review has revealed that the Mini-Cog test has an excellent sensitivity of 0.91 and an excellent specificity of 0.86 to detect dementia [111].

The 6MWT was developed for the measurement of aerobic capacity based on the maximum distance that individuals can walk in 6 min [112]. Assistive devices or bracing are allowed to be used and need to be documented. It has good to excellent test-retest reliability (ICC ≥ 0.76) [113]. The BHR test evaluates the endurance performance of the calf muscle, the triceps surae [114], and is performed in a standing position. Participants must stand barefoot, use the dominant hand on the wall to support themselves, and keep the elbow semi-flexed. First, participants do a full range of plantarflexion, and the examiner marks the maximum height at the participant’s head on the wall. Then, a marker is placed at the maximum height. After that, participants perform a full range plantarflexion repetitively as fast as possible, and their heads are supposed to touch the marker with each repetition. The maximum number of plantarflexions and the time taken to voluntary fatigue were recorded.

### 2.6. Statistical Analysis

In case of cerebral oxygenation measures, we applied second-level group statistical models using the NIRS Brain AnalyzIR Toolbox [104] where first an n-way ANOVA using factors, groups (active healthy, sedentary healthy, sedentary T2DM pre, sedentary T2DM post) and conditions (Mini-Cog step1, Mini-Cog step2, Mini-Cog step3), was applied (‘nirs.modules.AnovaN’). Moreover, we applied n-way ANOVA using factors, groups (sedentary T2DM pre, sedentary T2DM post) and conditions (Mini-Cog step1, step2, step3) to determine the 2-month moderate intensity exercise intervention effects in sedentary older adults with diabetes and cognitive impairment. The significance level was set at 0.05.

In case of muscle oxygenation measures, we applied Wilcoxon rank sum test that is a nonparametric test and was performed on the SmO2 drop and the SmO2 recovery speed to determine the 2-month moderate intensity exercise intervention effects in sedentary older adults with diabetes and cognitive impairment. The statistical analyses were performed in the software SPSS 28.0. The significance level was set at 0.05.

In case of cognitive and physical function tasks, we applied a Wilcoxon rank sum test that is a nonparametric test and was performed on the BHR repetitions and 6MWT distance to determine the 2-month moderate intensity exercise intervention effects in sedentary older adults with diabetes and cognitive impairment. The statistical analyses were performed in the software SPSS 28.0. The significance level was set at 0.05.

## 3. Results

**Figure 5:**
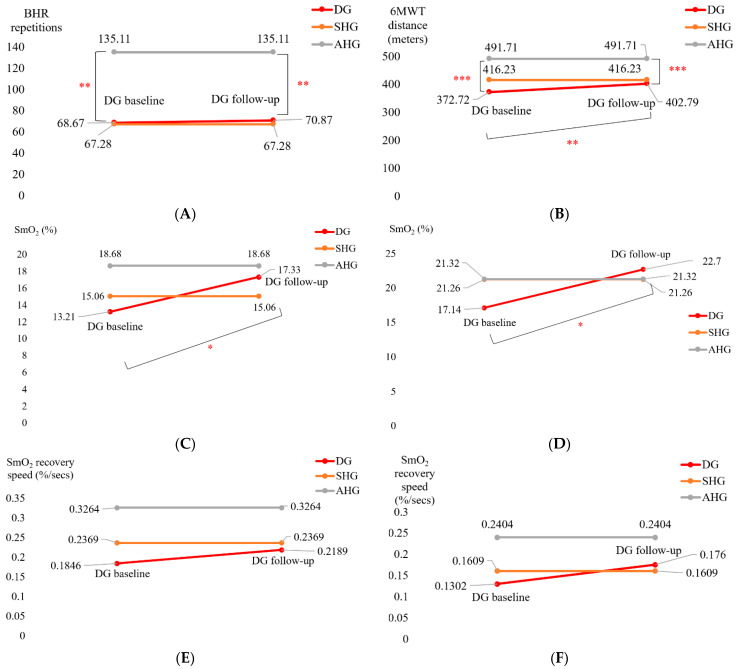
Comparison of pre- and post-intervention measurements using Wilcoxon rank sum test. (**A**) BHR repetitions comparisons. (**B**) 6MWT distance comparisons. (**C**) SmO2 drop during BHR comparisons. (**D**) SmO2 drop during 6MWT comparisons. (**E**) BHR SmO2 recovery rate comparisons. (**F**) 6MWT SmO2 recovery rate comparisons. “DG” is the T2DM Intervention group, “SHG” is the sedentary healthy Control group, “AHG” is the active healthy Control group. * *p* < 0.05, ** *p* < 0.01, *** *p* < 0.001.

The mean score of the adherence rate for the 21 participants in the T2DM Intervention group who adhered to the 2-month exercise, which was facilitated by biweekly phone calls, was 89.14% (SD = 21.23) based on the phone calls and pedometer monitoring. The 2-month moderate intensity exercise intervention in sedentary older adults with diabetes and cognitive impairment did not significantly affect the BHR repetitions at the post-intervention follow-up from pre-intervention baseline which was still significantly (** *p* < 0.01) lower than that of the active healthy Control group—see Figure 5A. However, the 6MWT distance significantly (** *p* < 0.01) improved in the T2DM Intervention group at post-intervention follow-up from pre-intervention baseline which was still significantly (*** *p* < 0.001) lower than that of the active healthy Control group—see Figure 5B. Figure 5C,D show the change in the SmO2 (%) drop in the T2DM Intervention group participants (“DG” in Figure 5) for BHR (Figure 5C) and the 6 min walk task (6MWT, Figure 5D) that significantly (* *p* < 0.05) changed at the post-intervention follow-up from the pre-intervention baseline. In fact, SmO2 (%) drop trended towards better than that of the sedentary healthy Control group (SHG in Figure 5) for BHR. Moreover, SmO2 (%) drop trended towards better than that of the active healthy Control group (AHG in Figure 5) as well as SHG for 6MWT (see Figure 5D). The SmO2 recovery rate also trended towards improvement after the 2-month moderate intensity exercise intervention from the pre-intervention baseline, as shown in Figure 5E,F, however, did not reach statistical significance (even at α = 0.05). Table 3 shows the SPSS 28.0 test results for the muscle oxygenation changes during BHR and the 6MWT.

**Figure 6:**
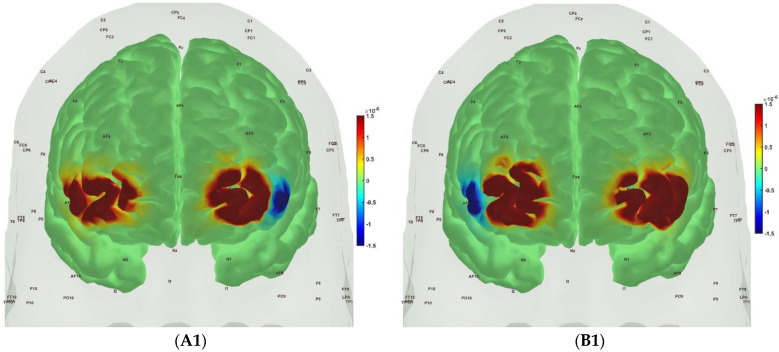

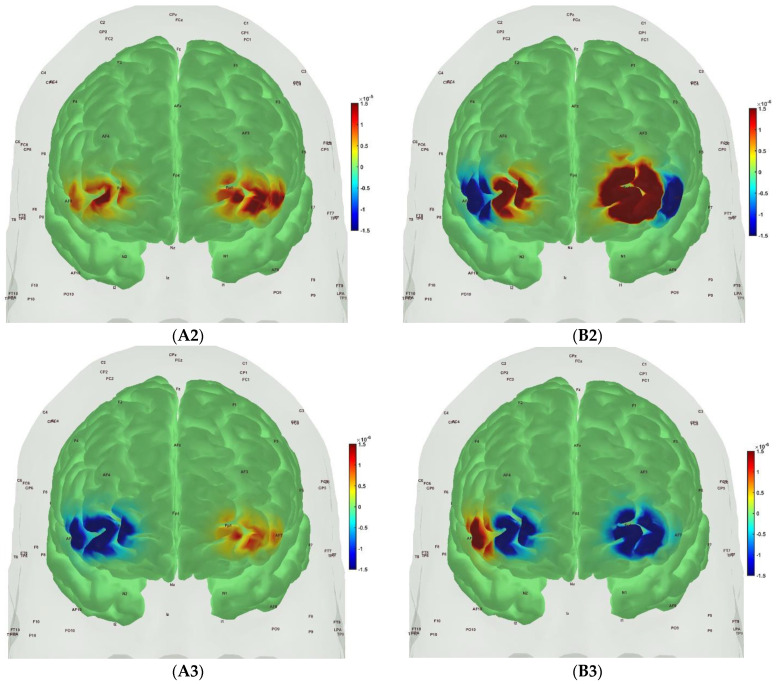
Prefrontal activation oxyHb (left panels, (**A1**–**A3**)) and deoxyHb (right panels, (**B1**–**B3**)) in the sedentary healthy Control group during (**A1**,**B1**): Mini-Cog word memory encoding (Mini-Cog step 1); (**A2**,**B2**): Mini-Cog clock drawing (Mini-Cog step 2); (**A3**,**B3**): Mini-Cog word recall (Mini-Cog step 3).

**Figure 7:**
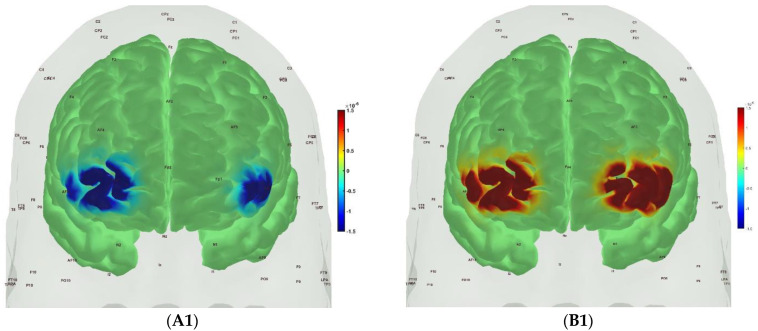

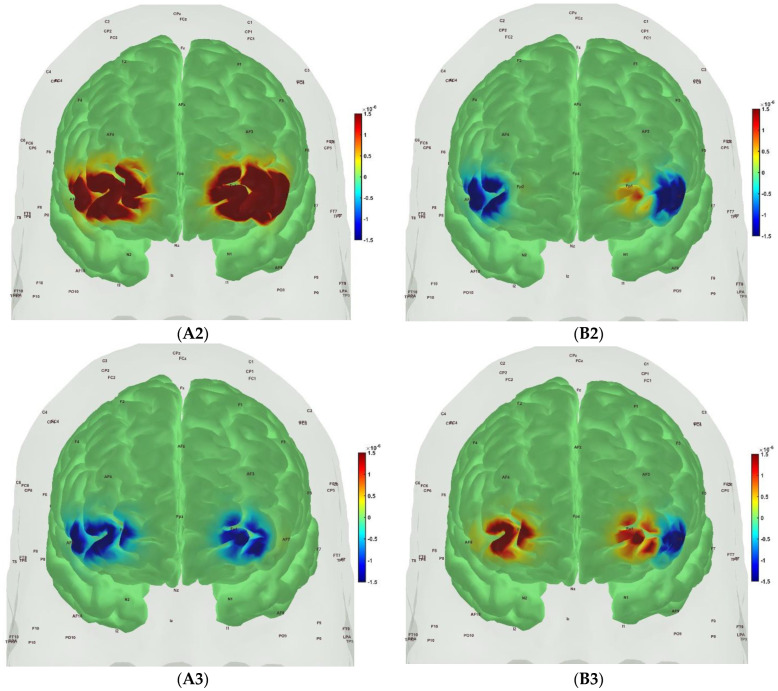
Prefrontal activation oxyHb (left panels, (**A1**–**A3**)) and deoxyHb (right panels, (**B1**–**B3**)) in the active healthy Control group during (**A1**,**B1**): Mini-Cog word memory encoding (Mini-Cog step 1); (**A2**,**B2**): Mini-Cog clock drawing (Mini-Cog step 2); (**A3**,**B3**): Mini-Cog word recall (Mini-Cog step 3).

**Figure 8:**
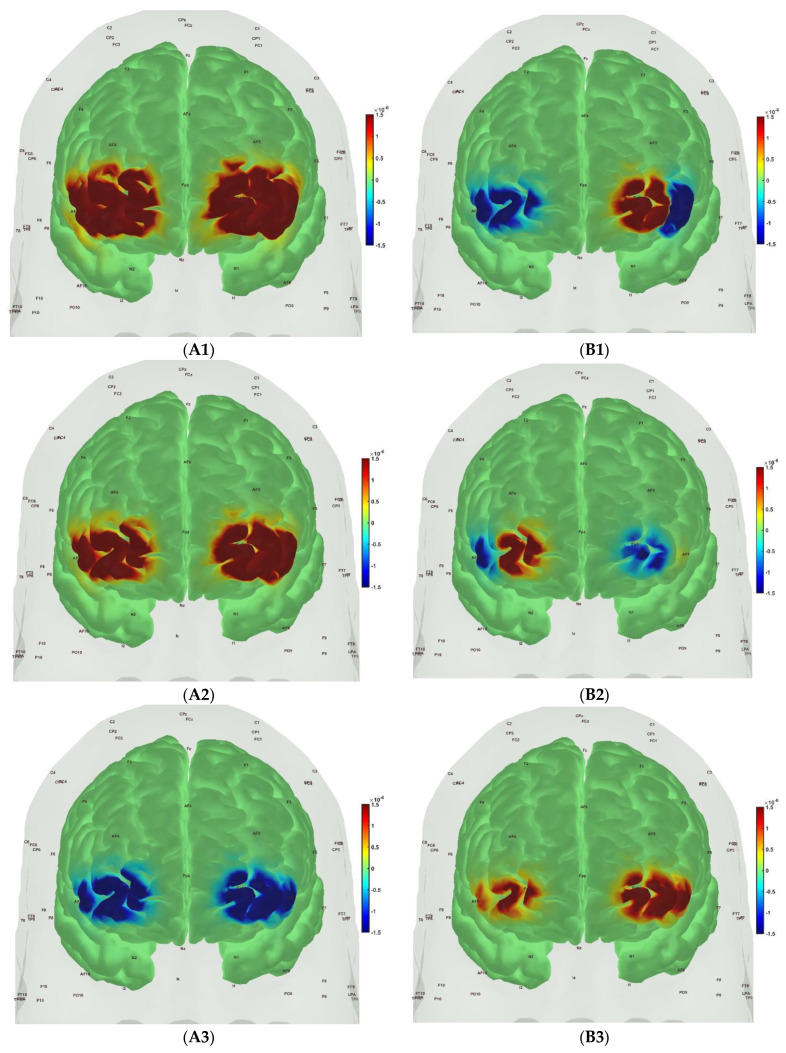
Prefrontal activation oxyHb (left panels, (**A1**–**A3**)) and deoxyHb (right panels, (**B1**–**B3**)) in the sedentary T2DM Intervention group at pre-intervention during (**A1**,**B1**): Mini-Cog word memory encoding (Mini-Cog step 1); (**A2**,**B2**): Mini-Cog clock drawing (Mini-Cog step 2); (**A3**,**B3**): Mini-Cog word recall (Mini-Cog step 3).

**Figure 9:**
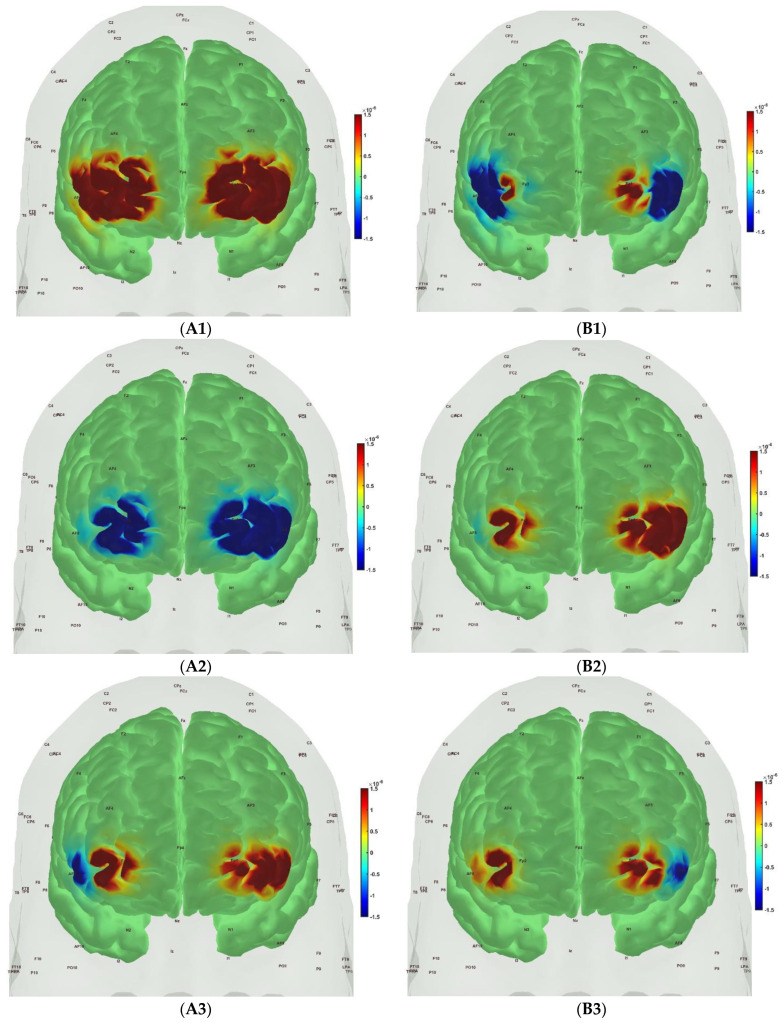
Prefrontal activation oxyHb (left panels, (**A1**–**A3**)) and deoxyHb (right panels, (**B1**–**B3**)) in the sedentary T2DM Intervention group at post-intervention during (**A1**,**B1**): Mini-Cog word memory encoding (Mini-Cog step 1); (**A2**,**B2**): Mini-Cog clock drawing (Mini-Cog step 2); (**A3**,**B3**): Mini-Cog word recall (Mini-Cog step 3).

Figure 6, Figure 7, Figure 8 and Figure 9 show the prefrontal activation (oxyHb in A and deoxyHb in B) from the AtlasViewer software based on the HRFs from the HOMER3 software computed during the Mini-Cog tasks (Mini-Cog step 1 in A1, B1; Mini-Cog step 2 in A2, B2; Mini-Cog step 3 in A3, B3). All the HRFs from the HOMER3 software are presented in the Supplementary Materials (Figure S1). Figure 6 shows the results from sedentary healthy Control group that showed bilateral superior frontal gyrus, dorsolateral, oxyHb, and deoxyHb activation during memory encoding and clock drawing tasks but only left Superior frontal gyrus, dorsolateral, and oxyHb activation and right Superior frontal gyrus, dorsolateral, oxyHb inactivation during the memory recall task. Then, Figure 7 shows the results from active healthy Control group that showed bilateral superior frontal gyrus, dorsolateral, and deoxyHb activation during the memory encoding task and oxyHb activation during clock drawing tasks and bilateral superior frontal gyrus, dorsolateral, and deoxyHb activation and oxyHb inactivation during the memory recall task. Here, oxyHb and deoxyHb activation may become positively correlated in the presence of systemic confounds and negative correlation between oxyHb and deoxyHb signals can be a characteristic of NVC [115]—see our HRFs in the Supplementary Materials (Figure S1). Notably, the superior frontal gyrus, dorsolateral, showed activation that is postulated to contribute to higher cognitive functions and particularly to working memory [116]. In prior works, Jahani et al. [89] showed the activation of the dorsolateral prefrontal cortex during memory encoding and recalling.

In the sedentary T2DM Intervention group, Figure 8 shows primarily negative correlation between oxyHb and deoxyHb activation at the bilateral superior frontal gyrus, dorsolateral, at the baseline (pre-intervention) that visibly changed at follow-up (post-intervention), as shown in Figure 9, with more similarity in prefrontal activation to Figure 7 for the active healthy Control group. N-way ANOVA in NIRS Brain AnalyzIR Toolbox [104] using factors, groups (active healthy, sedentary healthy, sedentary T2DM pre, sedentary T2DM post) and conditions (Mini-Cog step1, Mini-Cog step2, Mini-Cog step3), showed a statistically significant (*p* < 0.05, q < 0.05) effect of the groups (active healthy, sedentary healthy, sedentary T2DM pre, sedentary T2DM post) on the prefrontal activation at the AAL regions [117], Frontal_Sup_R and Frontal_Sup_L, based on both oxyHb and deoxyHb changes during Mini-Cog test—see Table 4.

Then, investigating the sedentary T2DM Intervention group for post-intervention changes from pre-intervention baseline, N-way ANOVA in NIRS Brain AnalyzIR Toolbox [104] using factors, groups (sedentary T2DM pre, sedentary T2DM post) and conditions (Mini-Cog step 1, Mini-Cog step 2, Mini-Cog step 3), showed a statistically significant (*p* < 0.05, q < 0.05) effect of the groups on the prefrontal activation (both, oxyHb and deoxyHb) at the AAL regions [117], Frontal_Sup_R, during Mini-Cog test—see Table 5. Then, when comparing groups (active healthy, sedentary T2DM post) and conditions (Mini-Cog step 1, Mini-Cog step 2, Mini-Cog step 3), N-way ANOVA in NIRS Brain AnalyzIR Toolbox [104] showed a statistically significant (*p* < 0.05, q < 0.05) effect of the groups on the prefrontal activation at the AAL regions [117], Frontal_Sup_R and Frontal_Inf_Tri_R, based on both oxyHb and deoxyHb changes during Mini-Cog test—see Table 6.

**Figure 10:**
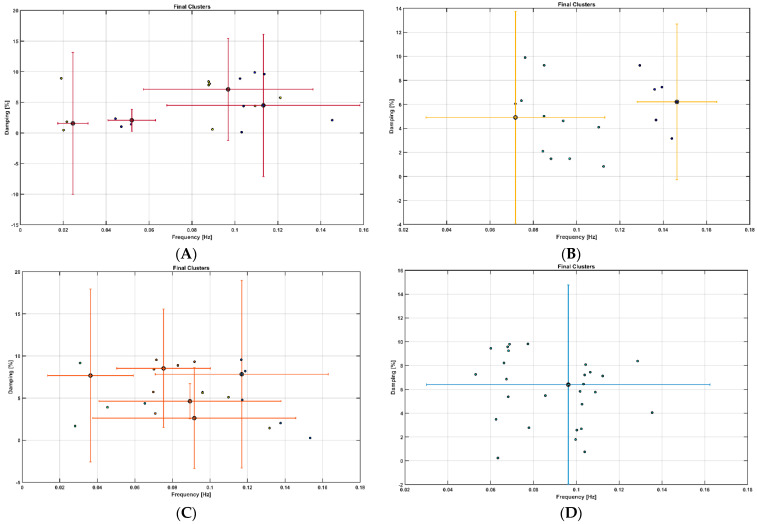
Results from OMA of the cognitive task-evoked hemodynamic responses where the cognitive load excitation of the prefrontal cortex was considered as realizations of the white noise processes. (**A**) Sedentary healthy Control group. (**B**) Active healthy Control group. (**C**) Sedentary T2DM Intervention group at baseline (pre-intervention). (**D**) Sedentary T2DM Intervention group at follow-up (post-intervention).

Spontaneous LFO of cerebral hemodynamics have been known in human adults [118] that can have neural as well as non-neural origin [119]. Therefore, the fNIRS measures of the effects of 2-month exercise programme in the sedentary T2DM Intervention group can have neural as well as non-neural origins that are both important in our opinion. For example, the effects of the 2-month exercise programme on the cognitive task-related autonomic cardiovascular arousal [120] and cerebral autoregulation [121] cannot be discounted due to the lack of short-separation regression [122]. Here, OMA provided insights into the importance of VLFO (<0.05 Hz) cluster during the Mini-Cog task that was present in the sedentary healthy Control group and the sedentary T2DM Intervention group at baseline but was missing after 2-month exercise programme in the sedentary T2DM Intervention group which was comparable to the active healthy Control group —see Figure 10. Moreover, the LFO (0.05–0.2 Hz) that can be NVC and myogenic influences [16,17] improved (increased power) after the 2-month exercise programme—see the power spectrum <0.1 Hz in the stabilization diagram shown in Figure S2 (supplementary materials). Here, VLFO (<0.02 Hz) can be endothelial related metabolic [16] and VLFO (>0.02 Hz and <0.05 Hz) can be neurogenic sympathetic while LFO (0.05–0.2 Hz) can be NVC and myogenic influences [16,17].

## 4. Discussion

Our study showed that the 2-month aerobic and resistance exercise programme influenced the VLFO (<0.05 Hz) cluster during a Mini-Cog test that was present in the sedentary T2DM Intervention group at baseline (pre-intervention) but was missing after 2-month exercise programme in the sedentary T2DM Intervention group—see Figure 10. The VLFO (>0.02 Hz and <0.05 Hz) clusters in the sedentary T2DM Intervention group at baseline (pre-intervention) and the sedentary healthy Control group can be related to vascular muscle and/or perivascular neurogenic regulation when other controls (vasomotor, chemical, and metabolic) become less dominant [123]. This aligns with the postulated cognitive task-related cerebral (in addition to systemic) autonomic cardiovascular arousal [120] due to cognitive load excitation—see also Table 5 and Table 6. Here, the cognitive task-related autonomic cardiovascular arousal [120] and cerebral autoregulation [121] effects cannot be discounted in T2DM [124]. We show in Figure S3 in the Supplementary Materials that the VLFO and LFO clusters were missing in the fNIRS short-separation channel measures [122] in the healthy Controls (N = 10) with only respiration-related ~0.3 Hz cluster [121] present while fNIRS long-separation channel measures have VLFO and LFO clusters as well as the respiration related cluster at ~0.3 Hz. Therefore, OMA of fNIRS long-separation channels captured all clusters of the cerebral hemodynamics [118] where a multi-stage clustering for automated OMA [108] using the Modal Toolkit (accessed on 12 June 2023 https://code.vt.edu/vibes-lab/modal-analysis) could separate the neural as well as the non-neural clusters [119] confirmed with the fNIRS short-separation channel measures. Here, various physiologically relevant frequency bands have already been identified in the literature: 0.6–2 Hz and 0.145–0.6 Hz are related to cardiac and respiratory function, respectively, 0.052–0.145 Hz is associated with smooth muscle cell activity, and 0.021–0.052 Hz may reflect smooth muscle autonomic innervation [125]. Then, in the setting of chronic hyperglycemia, abrupt changes in glycemic control can lead to small fiber neuropathy in patients [126] that may primarily affect 0.021–0.052 Hz oscillations. Then, the low oscillatory frequency (0.01–0.02 Hz) at capillaries may reflect vessel wall interaction under Fahræus–Lindqvist effect [127], i.e., the nonlinear dependence of apparent blood viscosity on hematocrit and vessel diameter thereby improving endothelial related metabolic interactions [16]. Here, blood viscosity and blood glucose are directly related: blood viscosity is lower in prediabetic subjects than in nondiabetic subjects with blood sugar levels that are high but within the normal range [128].

**Figure 11:**
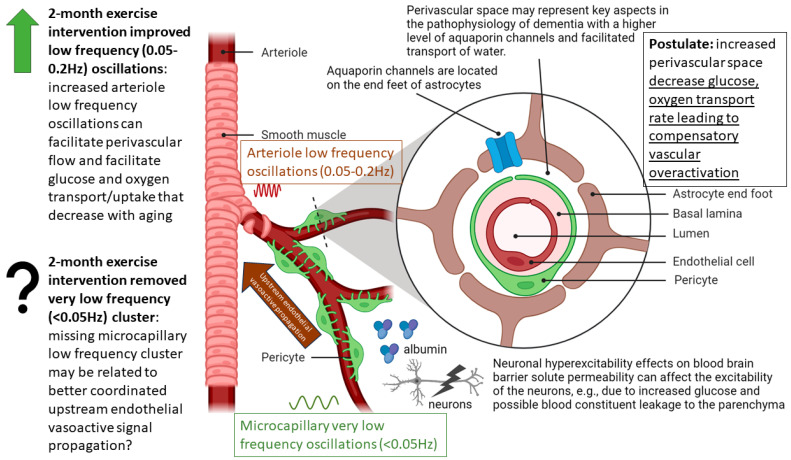
Summary of the mechanistic understanding of our OMA results. The ‘green arrow’ shows expected improvement in the LFO (0.05–0.2 Hz) due to 2-month exercise intervention; however, the ‘question mark’ shows that the effects on the VLFO (<0.05 Hz) need investigation vis-à-vis coordination of the microcapillary dilatations in VLFO with the arteriole dilatations in LFO via upstream endothelial vasoactive propagation and its communication with the vascular smooth muscle cells.

A statistically significant (*p* < 0.05, q < 0.05) effect of the 2-month exercise programme on the prefrontal activation (both, oxyHb and deoxyHb) at the AAL regions [117], Frontal_Sup_R, during the Mini-Cog task was found—see Table 5—based on post-intervention changes to pre-intervention baseline using N-way ANOVA in the NIRS Brain AnalyzIR Toolbox [104]. Frontal_Sup_R is the Right Superior frontal gyrus, dorsolateral, is a node of the dorsal and salience-ventral attention networks [31] thought to contribute to proactive control of impulsive response [129]. Figure 9 shows the average prefrontal activation during the Mini-Cog steps where Figure S1 in the Supplementary Materials provides further insights based on HRFs and shows a decreased oxyHb activation post-intervention than the pre-intervention baseline in Figure S1 (D) T2DM intervention group pre-intervention HRFs, and Figure S1 (E) T2DM intervention group post-intervention HRFs. Therefore, the 2-month exercise programme in the sedentary T2DM Intervention group addressed vascular overactivation which can be compensatory in T2DM due to neurometabolic and NVC dysfunction [31]. Then, when comparing active healthy and sedentary T2DM post for the conditions (Mini-Cog step1, Mini-Cog step2, Mini-Cog step3), N-way ANOVA in NIRS Brain AnalyzIR Toolbox [104] showed statistically significant (*p* < 0.05, q < 0.05) effect on the prefrontal activation at the AAL regions [117], Frontal_Sup_R and Frontal_Inf_Tri_R, between the active healthy versus the sedentary T2DM post-intervention groups based on both oxyHb and deoxyHb changes during Mini-Cog test—see Table 6. Again, Figure S1 in the Supplementary Materials provided further insights based on HRFs and show a decreased oxyHb activation in active healthy control group than the sedentary healthy control group in Figure S1 (B) Sedentary healthy control group HRFs. (C) Active healthy control group HRFs. Notably, the HRF shapes are different between the healthy control group (Figure S1B,C) and the T2DM intervention group (Figure S1D,E). Here, the effects of cognitive task-related autonomic cardiovascular arousal [120] at task onset cannot be discounted in the T2DM intervention group that can be related to dysfunctional vascular muscle, perivascular regulation [123,124]. In Figure 11, we present mechanistic understanding of the short 2-month aerobic and resistance exercise programme that is postulated to have influenced cerebral blood flow regulation via vasomotor, chemical, and metabolic controls that became more dominant [123]. Specifically, the VLFO (>0.02 Hz and <0.05 Hz) clusters in the sedentary T2DM Intervention group at baseline (pre-intervention) and the sedentary healthy Control group may be related to poor coordination of the microcapillary dilatations with the arteriole dilatations (via upstream endothelial vasoactive propagation and its communication with the vascular smooth muscle cells—see Figure 11) subserving NVC dysfunction which needs further investigation [16].

Muscle NIRS results supported the metabolic effect of 2-month aerobic and resistance exercise programme, as shown in Table 3, where the statistical test results show significant change in the SmO2 (%) drop in the T2DM Intervention group participants at the post-intervention follow-up for BHR and 6MWT tasks. This muscle NIRS changes aligned with the 6MWT distance that significantly (** *p* < 0.01) improved in the T2DM Intervention group at post-intervention follow-up from pre-intervention baseline—see Figure 5B. We have shown earlier reduced muscle oxidative capacity based on SmO2 changes during and after short exercise in older adults with obesity where active obese group’s oxidative capacity was similar to the inactive non-obese group, although lower than the active non-obese group [15]. Here, exercise-induced adaptations may include an altered profile of secreted proteins from skeletal muscle and adipose tissue [130] where adiposity in the skeletal muscle may be a risk factor for cognitive decline [131]. So, our study added evidence of impaired oxidative capacity in older adults with T2DM that needs combined strategy with obesity [132] with 150+ minutes/week moderate-intensity exercise and monitored with muscle oxygenation changes [15]—see Figure S4 in the Supplementary Materials for our exercise recommendation.

Limited evidence shows that exercise can reverse brain overactivation in older adults, e.g., a study reported decreased brain activation in related cortical regions during a semantic memory-related task among older adults with mild cognitive impairment after a 12-week walking intervention [133]. Another study demonstrated that prefrontal brain activation was reduced after a combined exercise program in frail older adults [134]. Our 2-month exercise programme outcomes align with these prior works. In the study conducted by Silveira-Rodrigues et al. [135], 31 sedentary middle-aged and older adults with T2DM were allocated to a combined aerobic and resistance exercise three times per week for 8 weeks or the control group. The 8-week aerobic and resistance exercise training improved attention/concentration, but not short-term memory in T2DM participants in the exercise group. In our study as well, the subjects improved the most in attention related Trail Making test Part A where post-intervention performance in the T2DM Intervention group was statistically similar to the Control group—see Figure S5 in the supplementary materials and Table 2. Short-term memory related Mini-Cog test and executive functioning related Trail Making test Part B performance post-intervention in the T2DM Intervention group remained statistically significantly different from the Control group—see Figure S5 in the supplementary materials and Table 2. These visual conceptual and visuo-motor tracking can be evaluated based on eye movement in response to a task battery [136]. Also, the authors [135] concluded that 8-week aerobic and resistance exercise training partially reversed the negative effects of T2DM possibly by the amelioration of metabolic dysfunction. Another study evaluated a 12-week pedometer-supported training intervention in 49 middle-aged and older adults with T2DM and found a significant improvement in the brain function [137]. In agreement to Silveira-Rodrigues et al.’s study [135], our results on the reduction of cognitive task related prefrontal overactivation is postulated to be due to better glucose utilization that is likely facilitated by the coordinated microcapillary and arteriole oscillations found from OMA—see Figure 10 and Figure 11. A longer duration, for example, 12 weeks intervention may have further improved prefrontal activation outcome, viz., Leischik et al. [137] study was conducted for 12 weeks. Congruent with our outcomes, a previous study found that motor planning in older adults likely relies on the over engagement of the prefrontal cortex and physical exercises can counteract the overactivity indicating possible metabolic and molecular effects [138]. Another study compared Kinect-based exergaming with combined exercise training effects in frail older adults [134,139]. In their study [134], 80% of the participants had normal cognition or mild dementia but not severe dementia. After 12 weeks of combined exercise, prefrontal overactivation decreased in both the groups during the Montreal Cognitive Assessment or The MoCA Test. Our pioneering study on OMA of fNIRS measures provided unique insights into the VLFO that was found missing after 2-month exercise intervention in the sedentary T2DM group that needs further investigation. Then, the decline of cognitive performance in T2DM was proposed to be also related to brain tissue impairments, including mitochondrial dysfunction [140], but is not well understood yet [141]. Since T2DM increases the risk of developing cognitive impairments, physical exercise can be used to reinforce antioxidative capacity, reducing oxidative stress in T2DM [140]. Oxidative phosphorylation produces energy or adenosine triphosphate (ATP) in the mitochondria in the presence of oxygen [142] and the ability of oxidative phosphorylation reflects oxidative capacity [143], where previous research has shown that individuals with T2DM had reduced oxidative capacity and low tolerance to exercise [144]. Exercise intervention has been identified as an effective way to reverse this situation and improve oxidative capacity, further enhance physical performance, and relieve fatigue [133,134]. Moreover, NO signaling participates in the mitochondrial respiration and biogenesis, where a decline in performance and an increase in fatigue among individuals with T2DM can be partly due to reduced oxidative phosphorylation [145] that may be ameliorated with aerobic exercise [146]. 

Recent evidence for white matter plasticity in older adults has been shown to be induced by aerobic walking and dance in healthy older adults [147] possibly due to improved bioenergetics. Studies on cognition in T2DM found that diabetes is pathophysiologically associated with cognitive decline [148,149,150]. Here, deficits in the structure and function of the prefrontal cortex are linked to reduced executive functions and poor glycemic control in T2DM [151]. In neurocognitive aging, the compensation hypothesis states that age-related overactivation is compensatory which is expressed by larger changes in oxyhemoglobin (oxyHb) with aging when processing cognitive tasks [152]. Several studies demonstrated that brain overactivation, especially in the dorsolateral prefrontal cortex while performing working memory tasks occurs in T2DM [153,154,155]. Here, the pattern of brain overactivation may be related to the dysfunction of the NVC cascade [36]. In the NVC cascade, the cerebrovascular reactivity to cognitive load may be dysfunctional where cerebral blood flow (CBF) increases due to brain activity [156,157,158]. This increase in blood flow at the neuronally activated brain areas is the physiological basis for most functional neuroimaging techniques. With the increase in CBF, the cerebral metabolic rate of oxygen (CMRO2) increases [159] that is also influenced by the oxygen extraction fraction (OEF) [160]. The increase in CBF is roughly twice that of the increase in CMRO2 to keep the partial pressure of the oxygen in the tissue at a steady level [161,162,163]. Then, the OEF can be altered in older adults with T2DM that can reduce task tolerance [164] where moderate-intensity training has been found helpful [165,166]. T2DM patients have been found to have dysregulated systemic hemodynamics during metaboreflex with an exaggerated blood pressure response and vasoconstriction in muscle [167] and brain [168]. Moreover, studies demonstrated reduced CBF with increasing age as one cause of cognitive impairment [169] as well as altered cerebral metabolism [170]. Compensation hypothesis has been proposed to explain brain overactivation with ageing and T2DM, where Zhang et al. [155] argued that strengthened regional activity in fronto-parietal networks may compensate for the declined working memory in T2DM subjects. NVC impairments (AD-like) may be subserving the compensation since an increased NVC has been found in the salience-ventral attention networks [31] linking T2DM and AD [32]. Moreover, people with mild cognitive impairment (MCI) seem to have abnormal increased medial temporal lobe activation early in the course of prodromal AD followed by a subsequent decrease as the disease progresses [171]. Therefore, overactivation seems like an initial compensation strategy in MCI patients to accomplish difficult cognitive tasks via increased NVC. Here, the compensation-related hypothesis states that some brain parts in older adults must work harder than young adults on similar tasks [152] and this deficit seems to be related to gray and white matter atrophy [172]. Indeed, vulnerability to NVC (AD-like) has been indicated differently for the default mode network when compared to the dorsal and salience-ventral attention networks [31]. Apart from the effects of age difference on brain overactivation, the other effect can be due to the nature and complexity of the cognitive tests, leading to overactivation in different regions of the prefrontal cortex. For newly diagnosed middle-aged T2DM subjects, a significant brain overactivation in the right dorsolateral prefrontal cortex, left middle/inferior frontal gyrus, and left parietal cortex were observed in the 2-back test [154]. However, overactivation was not detected during the 0-back and 1-back tests when compared with healthy controls [154], which indicates that the complexity of the task to evoke cognitive load excitation is important. In our current study, the clock drawing task evoked stronger prefrontal activation than the word memory encoding and recall tasks. The comparison of the two studies indicates that the cognitive task needs to evoke cognitive load excitation which can be achieved better with progressively higher n-back task [154] than the Mini-Cog word memorization task used in the current study. Then, cognitive load can significantly reduce blood glucose, such that the amount of cognitive load associated with task performance is an index of its sensitivity to glucose [173]. Here, the capacity limits on cognitive load may be explained by the limitations on the cellular metabolic energy that can be measured with broadband NIRS [174]. In fact, in a large cohort study [175], younger age at onset of diabetes increased the risk of subsequent dementia where AD has been called “type 3 diabetes” due to insulin resistance [3] where mitochondrial dysfunction and reduced ATP supply has been implicated [176]. Then, conditions of reduced ATP supply can lead to [Ca^2+^] overload in both neurons and astrocytes, leading to cellular stress, activation of lipases, and activation of microglia, which can eventually lead to synaptic loss in dementia syndromes [177]. To address mitochondrial dysfunction and reduced ATP supply, photobiomodulation can be applied to increase cytochrome c oxidase (CCO) activity [178] for the regulation of oxidative phosphorylation [179,180]. To delve further into the underlying mechanisms, an in vitro subject-specific brain organoid model, also described by Karanth et al. [181], can be subjected to oxygen glucose perturbations for detailed mechanistic studies. However, it is important to note that the current brain organoid model [181] does not replicate NVC. The incorporation of vascularized organoids, as suggested by Zhao et al. [182], may provide a viable avenue for studying NVC for individualized dosing. For individualized dosing, model predictive control of photobiomodulation can be elucidated with multimodal imaging [183] in 3D cultured brain organoids [184], and human model for system identification [185,186]. Our in vitro brain organoid study [181], accessed on 12 June 2023 https://neuromodec.org/nyc-neuromodulation-online-2020/P18.html, revealed significant findings of photobiomodulation effects, e.g., on the pH in the organoid tissue while simultaneously causing a decrease in the electrophysiological spectral exponent associated with the excitatory-inhibitory balance [187]. These preliminary results hold importance for future research on non-pharmacological therapeutics to address the neuronal hyperexcitability and the capacity limits of cellular metabolism [174] in T2DM and AD that can be based on individualized phase zero studies [188] using brain organoid platform [181]. This platform can incorporate a dual polymer sensor embedded within the Matrigel matrix, allowing real-time monitoring of glucose and oxygen levels [189] during mitochondrial photobiomodulation. This monitoring can facilitate the investigation of the neurometabolic dose/response relationship, enabling personalized delivery of treatment [18]. Moreover, mechanistic investigation of gene-environment interactions can be accomplished by utilizing a subject-specific brain organoid model derived from human-induced pluripotent stem cells (iPSCs), which enables the testing of optical theranostics [181].

In older adults with T2DM, genes encoding proteins of oxidative metabolism have been shown to be affected, and there were findings of decreased resting mitochondrial activity [190,191,192,193,194]. Low transport rate [195] of glucose, oxygen [196], and insulin [197] across increased perivascular space (see Figure 11) can switch the local parenchymal metabolism from glycolysis to fatty acid oxidation, mainly in the astrocytes lining the perivascular space—see Figure 11, and women have been found to be at an increased risk [198]. A glial upregulation of fatty acid metabolism to compensate for neuronal glucose hypometabolism in AD has been suggested which then correlates with amyloid and tau pathology [198]. So, we postulate that the hemodynamic overactivation may be subserved by hypometabolism, characterized by decreased brain glucose utilization, that is a vicious circle in neurodegenerative diseases [199]. Here, vessels with astrocyte-neuron metabolic cooperation are crucial [200] that can be affected by hyperglycemia impacting astrocyte energy metabolism and functional phenotype [201] leading to type 3 diabetes—a term proposed for AD [202]. Then, compensation by neuronal hyperexcitability will lead to elevated astrocytic [Ca^2+^] that will suppress arteriolar [Ca^2+^] oscillations [33] which may be identified with OMA. In fact, brain glucose hypometabolism is a prominent feature of AD [203], which may explain brain overactivation with ageing, where VLFOs (specifically, 0.02–0.07 Hz) and LFOs (specifically, 0.07–0.2 Hz) are known to be reduced in the older adults compared to the young during task performance [204]. Our prior work found a drop in the oscillatory power in the 0.01–0.02-Hz frequency band during the Mini-Cog assessment [205]; this drop was more pronounced in older participants with T2DM than in age-matched subjects that were normoglycemic [16]. Here, a mechanistic understanding of brain energy metabolism is essential vis-a-vis 0.01–0.02 Hz oscillations [127] which may explain the basis of “diabetic brain fog.” For example, the maximal exercise capacity has been found to be positively associated with microvascular glycocalyx thickness at baseline [68] and deteriorated glycocalyx could initiate cardiovascular disease pathology [69]. Regular exercise intervention can improve the spontaneous activity in the vascular cells with reduced vessel stiffness (see Figure 11) where a mechanistic understanding can allow development of transcranial electrical stimulation (tES) to modulate LFOs in the early stages [206]. 

In future work, grey-box modeling and causal inference from portable fNIRS measures [207] in response to tES [208] may capture the changes in the neurovascular (including small blood vessels) and neurometabolic (including mitochondria) system [209] in T2DM for model-predictive [210] individualized dosing. tES has been shown to reduce blood glucose in men through an insulin-independent mechanism that relates to brain energy metabolism [211]. We have investigated the transient initial (0–150 s) total hemoglobin response to transcranial direct current stimulation (tDCS) [19] after removing the oscillatory nonlinear calcium dynamics during myogenic smooth muscle activity in the frequency range of 0.05–0.2 Hz, including the ∼0.1-Hz hemodynamic oscillations in the fNIRS time series. Here, we investigated VLFO (0.01 to 0.05 Hz), which are crucial in small vessel function and may be dysfunctional in T2DM. In our study on 19 elderly (60 years and older) with T2DM and 38 age-matched controls [16], we found a significantly lower relative power in the 0.021–0.052 Hz frequency band in elderly subjects with T2DM during the Mini-Cog three-item recall test [126]. Then, ∼0.1-Hz hemodynamic oscillations can be related to the synchronization of the intermittent release of calcium within vascular mural cells, including smooth muscle cells, where contractile mural cells are known to generate spontaneous calcium transients. Investigation of coupled steady-state vessel oscillations in the low-frequency (≤0.1 Hz) range coupled with electroencephalogram (EEG) band power [212] in patients with early-stage AD with and without T2DM is motivated by prior works that found a cross-correlation between log (base 10)-transformed EEG band power (0.5–11.25 Hz) and fNIRS O2Hb signals in that low-frequency (≤0.1 Hz) range. Then, longer duration (>3 min with 1 mA [213]) tDCS application can have polarity specific effects [214] that can modulate the cortical excitability, likely by the potassium (K^+^) ions [215,216] that are released and accumulate in the vicinity of capillaries. The potassium (K^+^) ions [215,216] in the vicinity of capillaries can be sensed by the capillary network with the inwardly rectifying K^+^ channel Kir acting as the sensor, which can then modulate the neurovascular system’s sensitivity [19,217]. Moreover, longer-duration tDCS, postulated to elevate extracellular K^+^, can decrease calcium activity mediated by the inward-rectifying potassium (Kir) channel in mural cells [217]. Besides Kir channels, voltage-dependent potassium channels, calcium-activated potassium channels, and ATP-activated potassium channels are also present in the mural cells and can interact with the dilatory stress-induced calcium transients. Here, tES effects on the contractile mural cells that encircle the precapillary sphincter [185] at the transition between the penetrating arteriole and the first-order capillary may be crucial for intracerebral microcapillary modulation [218] by tES. Then, tDCS modulated brain activity and neuronal glucose metabolism are linked [219] to longer duration (>3 min with 1 mA [213]) application that can lead to the accumulation of local metabolites which can influence CBF regulation via changes in the vasomotor, chemical, and metabolic controls [103,123]. We postulate that the subject-specific tES dose−response can be captured by the neurovascular (between the hemodynamic fNIRS signal with the EEG band power) and neurometabolic (between the CCO fNIRS signal with the EEG band power) coupling for model predictive control of tES [18,220]. Here, a physiologically detailed NVC model [19], showed that all pathways in the neurovascular unit evoked steady-state vessel oscillations, which is expected from the experimental literature [221]. Moreover, the EEG band power oscillations and the vasomotion can entrain each other [221], which may be modulated by model predictive control of tES [18,212] that can facilitate perivascular transport and paravascular clearance [18,222].

The current study has several strengths as well as limitations. One limitation is the study design, as it lacked a true control group of individuals with T2DM subjects without any intervention. This was due to time constraints, funding shortages, and the ongoing COVID-19 pandemic. Instead, the study compared T2DM participants with healthy controls measured once. However, if the healthy controls had been measured twice during the COVID-19 pandemic, their physical activity levels would likely have declined, potentially biasing the outcomes in favor of the study. In the current study, the T2DM intervention group had an improvement in the SmO2 (%) drop compared to the active healthy group as well as the sedentary healthy group during the 6MWT. The study also lacked T2DM participants in the physically active category, limiting the analysis controlling for physical activity levels. Then, the intervention and control groups were only matched based on age and sex, with no further matching criteria. Moreover, our targeted subjects 60 years or older did not aim for the generalizability of the findings to younger T2DM populations or individuals without T2DM with cognitive impairments. Another limitation relates to the self-reporting of diet. Although most participants reported no changes in their diet, the accuracy of self-reporting was not verified. This is important because diet can impact insulin control, which is relevant to the study’s objectives. The third limitation is the high dropout rate of 34.7%. Various reasons accounted for the dropout, including injuries, failed screenings, COVID-19 infections, health issues, personal schedule changes, and family reasons. The high dropout rate resulted in a small sample size, which may have increased the likelihood of Type II errors (failure to find a difference when one exists) and reduced statistical power. Additionally, the study lacked follow-up with participants longer term after the two-month exercise intervention and their quality of life, which could have provided valuable insights into adherence to the exercise program and its long-term effectiveness. It would have been beneficial to explore the participants’ ability and quality of life to motivate the continuation of higher intensity exercise after improving their activity levels and performance in 2 months. Another challenge faced during the study was eliminating systemic noise from recorded data due to unavailability of short separation channel measures in most of the T2DM subjects. Therefore, we verified the negative correlation between oxyHb and deoxyHb signals as a characteristic of NVC—see the HRFs in Figure S1 and the OMA clusters in Figure S3 of the Supplementary Materials. Despite these limitations, the study has strengths. The completion rate for the 2-month exercise program was high, with participants completing an average of 89.14% of exercise sessions. This indicates that the exercise program was practical, doable, and well tolerated, even during the pandemic. The program was safe, required minimal equipment (ankle weights), and did not require supervision. The exercise instructions were easy to understand, making them suitable for older adults with cognitive decline. The study provided innovative evidence on brain overactivation among older adults with T2DM, supporting the compensatory theory. It demonstrated that the 2-month combined exercise intervention effectively reduced brain overactivation and contributed to improved cognitive function. Here, OMA results indicated an exercise-related effect on VLFO (<0.05 Hz) cluster that can be postulated to be related to better microvascular function. Furthermore, the study personalized exercise duration and interval based on muscle oxygenation during physical tasks, leading to improvements in muscle oxidative capacity in just two months. This finding has clinical implications for physical therapists, as targeting muscle oxygenation changes during physical tests and utilizing the information to prescribe proper exercise dose in metabolic syndrome that can enhance physical performance. 

In summary, this study had limitations in terms of study design, lack of control group, limited analysis of physical activity levels, self-reporting of diet without verification, high dropout rate, and absence of long-term follow-up. However, it also demonstrated practicality and effectiveness of our 2-month exercise program, OMA provided valuable insights into brain and muscle response to the 2-month exercise program, and offered portable muscle imaging for personalized exercise recommendations that can be based on muscle oxygenation changes for future clinical trials. Indeed, adiposity accumulating in the skeletal muscle in metabolic syndrome can be an important risk factor for cognitive decline. 

## Figures and Tables

**Table 1:** Demographic information.

Characteristics	T2DM Intervention Group at Baseline N (%) or Mean (Standard Deviation)	Sedentary Healthy (SH) Control Group N (%) or Mean (Standard Deviation)	Active Healthy (AH) Control Group N (%) or Mean (Standard Deviation)	F or χ^2^
Age	66.1 (4.5)	66.6 (4.2)	65.9 (4.2)	F = 0.055 (*p* = 0.947)
BMI	34.8 (4.8)	33.3 (5.3)	26.1 (4.3) ***	F = 18.775 *** (*p* < 0.001) T2DM vs. AH
Sex				
Male	10 (50.0%)	10 (50.0%)	10 (50.0%)	χ^2^ = 1.0
Female	10 (50.0%)	10 (50.0%)	10 (50.0%)	(*p* = 1.000)
Ethnicity				
Hispanic	3 (15.0%) *	0	0	
Non-Hispanic	17 (85.0%)	100 (100.0%)	100 (100.0%)	χ^2^ = 6.316 * (*p* = 0.043)
Race				
White	14 (70.0%)	17 (85.0%)	19 (95.0%)	
Black/African American	5 (25.0%)	1 (5.0%)	0 (0.0%)	χ^2^ = 8.260 (*p* = 0.083)
Other	1 (3.0%)	2 (10.0%)	1 (5.0%)	
Living status				
Alone	4 (20.0%)	9 (45.0%)	5 (25.0%)	χ^2^ = 3.333
With someone	16 (80.0%)	11 (55.0%)	15 (75.0%)	(*p* = 0.189)
Marital Status Single Married Widowed Separated/Divorced	2 (10.0%) 15 (75.0%) 1 (5.0%) 2 (10.0%)	5 (25.0%) 9 (45.0%) 2 (10.0%) 4 (20.0%)	6 (30.0%) 11 (55.0%) 1 (5.0%) 2 (10.0%)	χ^2^ = 5.100 (*p* = 0.513)
Home Ownership				
Rent	2 (10.0%)	2 (10.0%)	2 (10.0%)	χ^2^ = 2.038
Own	17 (85.0%)	18 (90.0%)	18 (90.0%)	(*p* = 0.729)
Other	1 (5.0%)	0 (0.0%)	0 (0.0%)	
Work Status				
Full Time	5 (25.0%)	7 (35.0%)	6 (30.0%)	
Part time	5 (25.0%)	3 (15.0%)	2 (10.0%)	χ^2^ = 3.333
Retired	9 (45.0%)	9 (45.0%)	12 (60.0%)	(*p* = 0.737)
Unemployed	1 (5.0%)	1 (5.0%)	0 (0.0%)	
Income				
Very Comfortable	4 (20.0%)	6 (30.0%)	10 (50.0%)	
Comfortable	14 (70.0%)	13 (65.0%)	9 (45.0%)	χ^2^ = 5.967
Uncomfortable	1 (5.0%)	1 (5.0%)	1 (5.0%)	(*p* = 0.427)
Not comfortable at all	1 (5.0%)	0 (0.0%)	0 (0.0%)	
Education				
High School	3 (15.0%)	2 (10.0%)	5 (25.0%)	
2 Year College	9 (45.0%)	8 (40.0%)	1 (5.0%)	
BS/BA	3 (15.0%)	5 (25.0%)	3 (15.0%)	χ^2^ = 12.261
MA/MS	4 (20.0%)	3 (15.0%)	8 (40.0%)	(*p* = 0.140)
More Advanced Degree	1 (5.0%)	2 (10.0%)	3 (15.0%)	

* *p* < 0.05, ** *p* < 0.01, *** *p* < 0.001 For Chi-squares, * indicates the standard residuals results ≥ 2.0 or ≤ −2.0.

**Table 2:** Cognitive performance comparisons between the participants in the sedentary T2DM Intervention group at baseline and the active and sedentary healthy Control group.

Characteristics	Intervention Group at Baseline Mean (Standard Deviation)	Control Group Mean (Standard Deviation)	Z	Cohen’s d
Mini-Cog	12.79 (2.1)	14.16 (0.9)	Z = 3.273 ** (*p* = 0.0005)	d = 0.967
Trail Making Part A (s)	39.55 (12.1)	30.94 (6.8)	Z = 2.548 ** (*p* = 0.006)	d = 0.972
Trail Making Part B (s)	93.45 (26.58)	69.08 (21.3)	Z = 3.293 *** (*p* < 0.001)	d = 1.053

* *p* <0.05, ** *p* < 0.01, *** *p* < 0.001.

**Table 3:** Muscle oxygenation changes during BHR and the 6MWT in the T2DM Intervention group at pre-intervention baseline and post-intervention follow-up.

Characteristics	Baseline M(SD)	Follow-Up M (SD)	t or Z	Cohen’s d
SmO2 drop during BHR test (%)	13.21 (7.5)	17.33 (11.6)	t = 2.185 * (*p* = 0.022)	d = −0.515
SmO2 drop during 6MWT (%)	17.14 (9.1)	22.70 (15.4)	t = 1.845 * (*p* = 0.041)	d = −0.435
BHR SmO2 recovery speed (%/s)	0.1846 (0.071)	0.2189 (0.107)	t = 1.714 (*p* = 0.052)	d = −0.404
6MWT SmO2 recovery speed (%/s)	0.1302 (0.087)	0.1760 (0.174)	t = 1.094 (*p* = 0.145)	d = −0.258

* *p* < 0.05, ** *p* < 0.01, *** *p* < 0.001.

**Table 4:** Results from N-way ANOVA in NIRS Brain AnalyzIR Toolbox [104] using factors, groups (active healthy, sedentary healthy, sedentary T2DM pre, sedentary T2DM post) and conditions (Mini-Cog step1, Mini-Cog step2, Mini-Cog step3) for the AAL regions [117] covered by fNIRS probe.

AAL Region	Source	Detector	Type	Factor	*p*	q
Frontal_Inf_Tri_R	1	1	‘hbo’	‘cond’	0.999999981	1
Frontal_Inf_Tri_R	1	1	‘hbo’	‘group’	0.083678248	0.205977227
Frontal_Inf_Tri_R	1	1	‘hbr’	‘cond’	0.947160368	1
**Frontal_Inf_Tri_R**	**1**	**1**	**‘hbr’**	**‘group’**	**0.000314435**	**0.001437418**
Frontal_Sup_R	3	1	‘hbo’	‘cond’	0.999987864	1
**Frontal_Sup_R**	**3**	**1**	**‘hbo’**	**‘group’**	**0.003211619**	**0.012846478**
Frontal_Sup_R	3	1	‘hbr’	‘cond’	1	1
**Frontal_Sup_R**	**3**	**1**	**‘hbr’**	**‘group’**	**1.97 × 10^−10^**	**3.15 × 10^−9^**
Frontal_Inf_Tri_L	5	2	‘hbo’	‘cond’	0.999999039	1
Frontal_Inf_Tri_L	5	2	‘hbo’	‘group’	0.064006828	0.170684874
Frontal_Inf_Tri_L	5	2	‘hbr’	‘cond’	0.994658711	1
**Frontal_Inf_Tri_L**	**5**	**2**	**‘hbr’**	**‘group’**	**1.82 × 10^−5^**	**9.70 × 10^−5^**
Frontal_Sup_L	7	2	‘hbo’	‘cond’	0.375322014	0.720721989
**Frontal_Sup_L**	**7**	**2**	**‘hbo’**	**‘group’**	**6.15 × 10^−7^**	**4.92 × 10^−6^**
Frontal_Sup_L	7	2	‘hbr’	‘cond’	0.999999845	1
**Frontal_Sup_L**	**7**	**2**	**‘hbr’**	**‘group’**	**3.44 × 10^−6^**	**2.20 × 10^−5^**

The AAL regions highlighted in **Bold** are significant at *p* < 0.05 and q < 0.05.

**Table 5:** Results from N-way ANOVA in NIRS Brain AnalyzIR Toolbox [104] using factors, groups (sedentary T2DM pre, sedentary T2DM post) and conditions (Mini-Cog step1, Mini-Cog step2, Mini-Cog step3) for the AAL regions [117] covered by fNIRS probe.

AAL Region	Source	Detector	Type	Factor	*p*	q
Frontal_Inf_Tri_R	1	1	‘hbo’	‘cond’	0.6481	1
**Frontal_Inf_Tri_R**	**1**	**1**	**‘hbo’**	**‘group’**	**0.00587**	**0.01879**
Frontal_Inf_Tri_R	1	1	‘hbr’	‘cond’	0.78319	1
Frontal_Inf_Tri_R	1	1	‘hbr’	‘group’	0.62103	1
Frontal_Sup_R	3	1	‘hbo’	‘cond’	0.97924	1
**Frontal_Sup_R**	**3**	**1**	**‘hbo’**	**‘group’**	**2.39 × 10^−6^**	**8.50 × 10^−6^**
Frontal_Sup_R	3	1	‘hbr’	‘cond’	1	1
**Frontal_Sup_R**	**3**	**1**	**‘hbr’**	**‘group’**	**2.15 × 10^−6^**	**8.50 × 10^−6^**
Frontal_Inf_Tri_L	5	2	‘hbo’	‘cond’	1	1
Frontal_Inf_Tri_L	5	2	‘hbo’	‘group’	0.7415	1
Frontal_Inf_Tri_L	5	2	‘hbr’	‘cond’	0.99998	1
Frontal_Inf_Tri_L	5	2	‘hbr’	‘group’	0.56073	1
Frontal_Sup_L	7	2	‘hbo’	‘cond’	1	1
**Frontal_Sup_L**	**7**	**2**	**‘hbo’**	**‘group’**	**7.59** **× 10^−8^**	**3.47** **× 10^−7^**
Frontal_Sup_L	7	2	‘hbr’	‘cond’	0.99781	1
Frontal_Sup_L	7	2	‘hbr’	‘group’	0.56618	1

The AAL regions highlighted in **Bold** are significant at *p* < 0.05 and q < 0.05.

**Table 6:** Results from N-way ANOVA in NIRS Brain AnalyzIR Toolbox [104] using factors, groups (active healthy, sedentary T2DM post) and conditions (Mini-Cog step1, Mini-Cog step2, Mini-Cog step3) for the AAL regions [117] covered by fNIRS probe.

AAL Region	Source	Detector	Type	Factor	*p*	q
Frontal_Inf_Tri_R	1	1	‘hbo’	‘cond’	0.99999259	1
Frontal_Inf_Tri_R	1	1	‘hbo’	‘group’	0.056004	0.17322566
Frontal_Inf_Tri_R	1	1	‘hbr’	‘cond’	1	1
**Frontal_Inf_Tri_R**	**1**	**1**	**‘hbr’**	**‘group’**	**0.00543628**	**0.04349027**
Frontal_Sup_R	3	1	‘hbo’	‘cond’	0.99999952	1
Frontal_Sup_R	3	1	‘hbo’	‘group’	0.01244236	0.05687937
Frontal_Sup_R	3	1	‘hbr’	‘cond’	1	1
**Frontal_Sup_R**	**3**	**1**	**‘hbr’**	**‘group’**	**8.41 × 10^−12^**	**1.35 × 10^−10^**
Frontal_Inf_Tri_L	5	2	‘hbo’	‘cond’	0.09426186	0.2320292
Frontal_Inf_Tri_L	5	2	‘hbo’	‘group’	0.06495962	0.17322566
Frontal_Inf_Tri_L	5	2	‘hbr’	‘cond’	0.97629652	1
Frontal_Inf_Tri_L	5	2	‘hbr’	‘group’	0.28616516	0.57233033
Frontal_Sup_L	7	2	‘hbo’	‘cond’	0.01672316	0.06689266
Frontal_Sup_L	7	2	‘hbo’	‘group’	0.96488565	1
Frontal_Sup_L	7	2	‘hbr’	‘cond’	1	1
Frontal_Sup_L	7	2	‘hbr’	‘group’	0.04105912	0.14598797

The AAL regions highlighted in **Bold** are significant at *p* < 0.05 and q < 0.05.

## Data Availability

The human data that support the findings of this study are available upon reasonable request. Due to privacy and ethical restrictions, the raw data cannot be publicly shared. However, interested researchers can contact the corresponding author to request access to the de-identified dataset for the purpose of further analysis and replication of the results.

## Supplementary Materials

The following supporting information can be downloaded at: https://www.mdpi.com/article/10.3390/brainsci13071099/s1, Figure S1: Averaged hemodynamic response function (HRF) of the experimental groups with solid line showing oxyhemoglobin concentration changes and the dotted line showing the deoxyhemoglobin concentration changes. Figure S2: Operational modal analysis (OMA) of near-infrared spectroscopy effects with maximum 30 modes for the power spectrum <0.1Hz in the stabilization diagram shown. Figure S3: Operational modal analysis (OMA) of near-infrared spectroscopy signals from long-separation and short-separation channels with maximum 30 modes for the power spectrum <0.1 Hz in the stabilization diagram shown for health control subjects (N = 10). Figure S4: Postulated indicators of the muscle oxygenation response—resting SmO2, SmO2 drip during exercise, SmO2 recovery after exercise. Figure S5: Cognitive test comparisons between pre-intervention baseline and post-intervention.
