# Supplementary material for: Operational Modal Analysis of Near-Infrared Spectroscopy Measure of 2-Month Exercise Intervention Effects in Sedentary Older Adults with Diabetes and Cognitive Impairment
Source: Brain Sci. 2023 Jul 20;13(7):1099. doi: 10.3390/brainsci13071099 (PMC10377417; doi:10.3390/brainsci13071099)
Supplement: Supplementary file 1 [file brainsci-13-01099-s001.zip › brainsci-2473557-SI.pdf]

Supplementary Materials

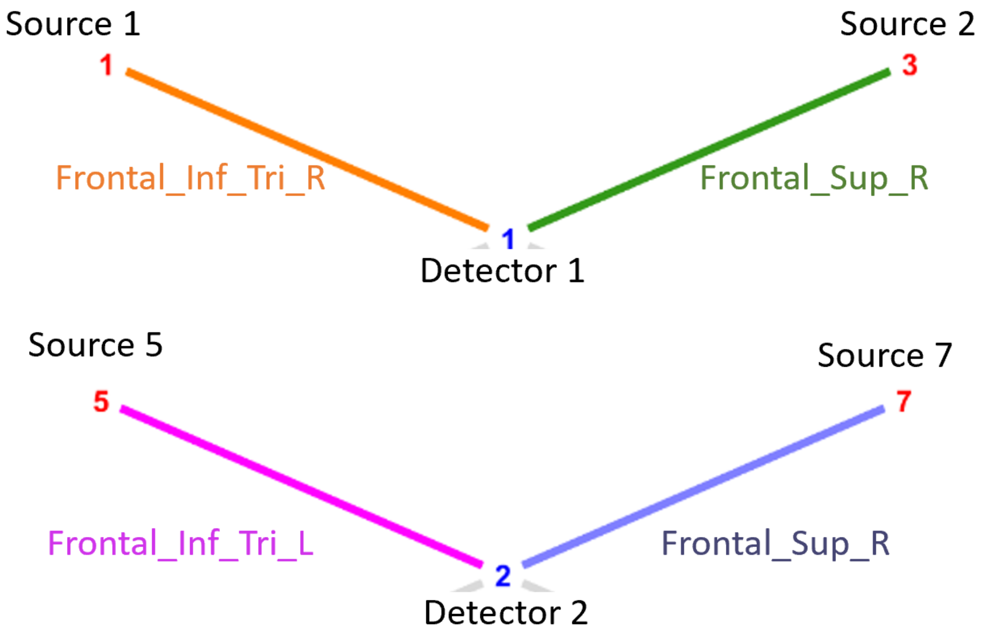

(A)

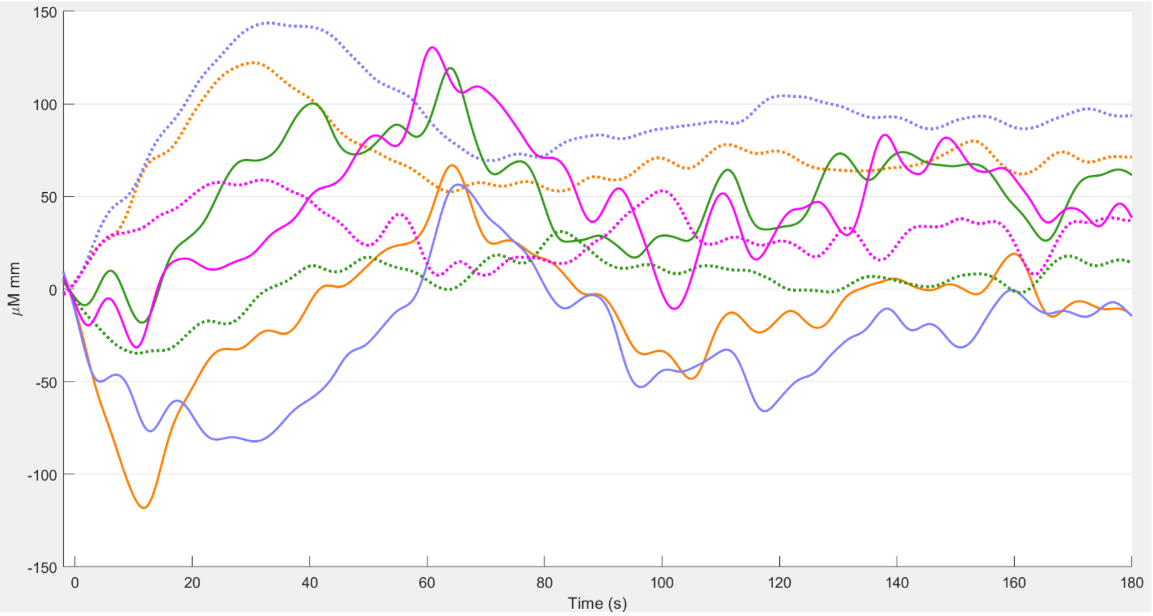

(B)

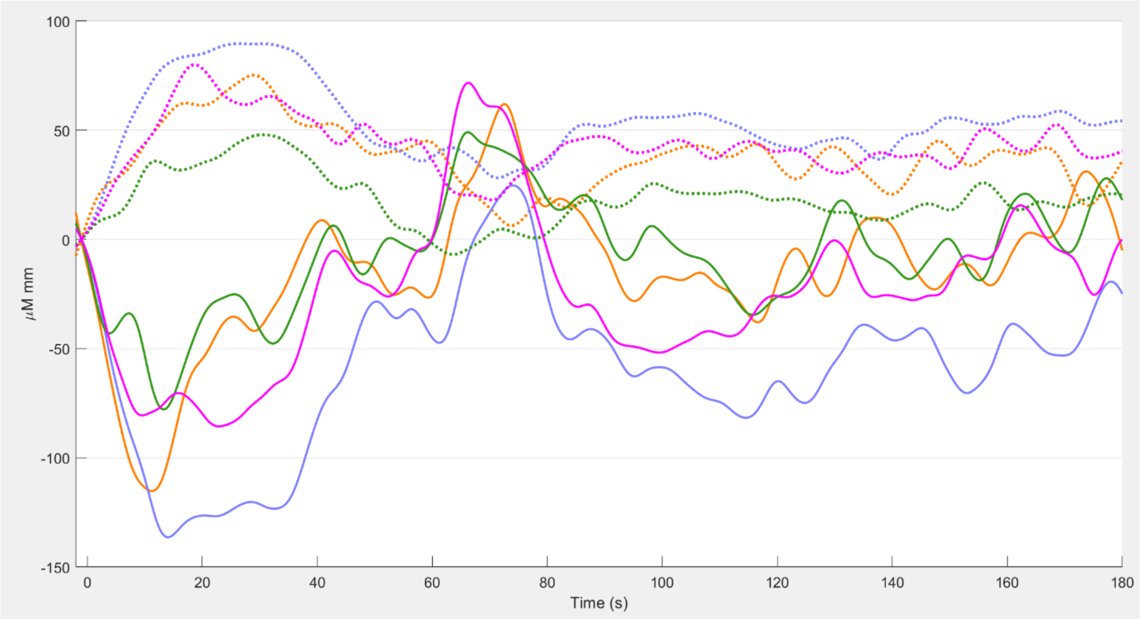
100
50
0
-50
-100
-150
μM mm
0
20
40
60
80
100
120
140
160
180
Time (s)

(C)

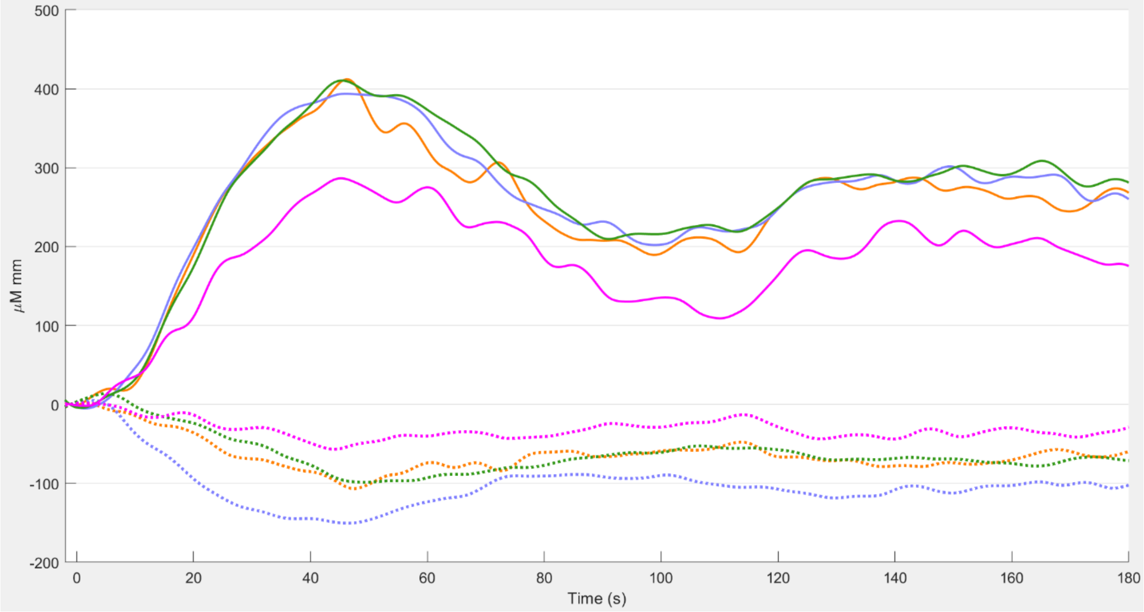
500
400
300
200
100
0
-100
-200
μM mm
0
20
40
60
80
100
120
140
160
180
Time (s)

(D)

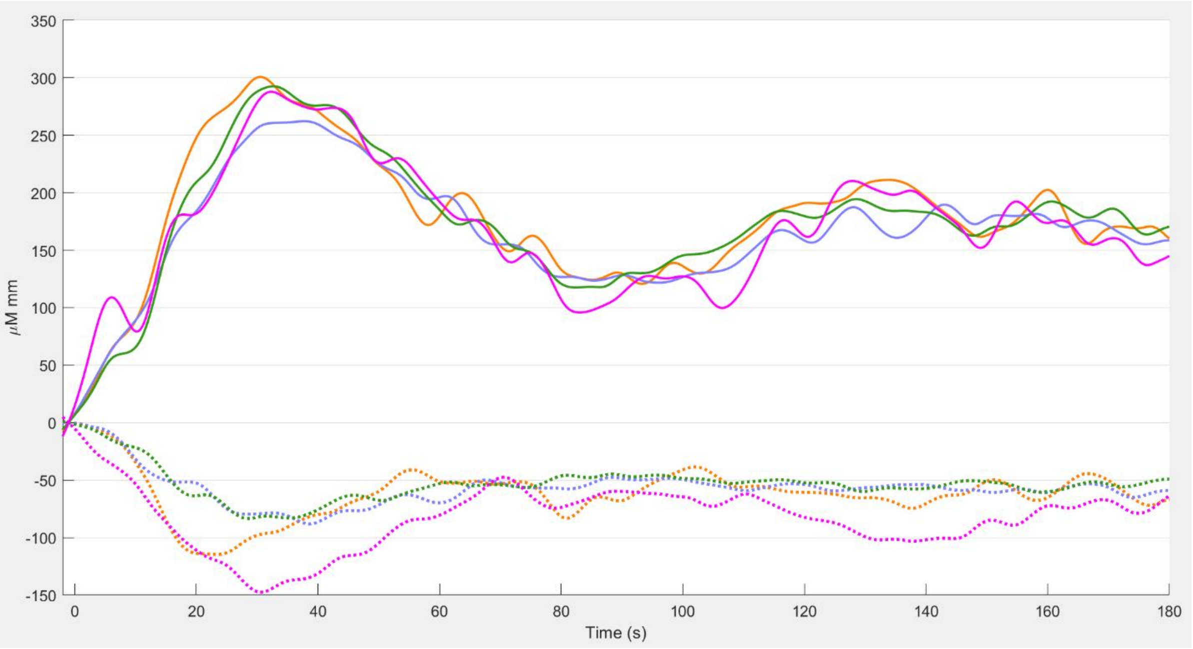

(E)

Figure S1. Averaged hemodynamic response function (HRF) of the experimental groups with solid line showing oxyhemoglobin concentration changes and the dotted line showing the deoxyhemoglobin concentration changes. (A) Optode montage with colors denoting different prefrontal brain regions of interest. (B) Sedentary healthy control group HRFs. (C) Active healthy control group HRFs. (D) T2DM intervention group pre-intervention HRFs. (E) T2DM intervention group post-intervention HRFs.

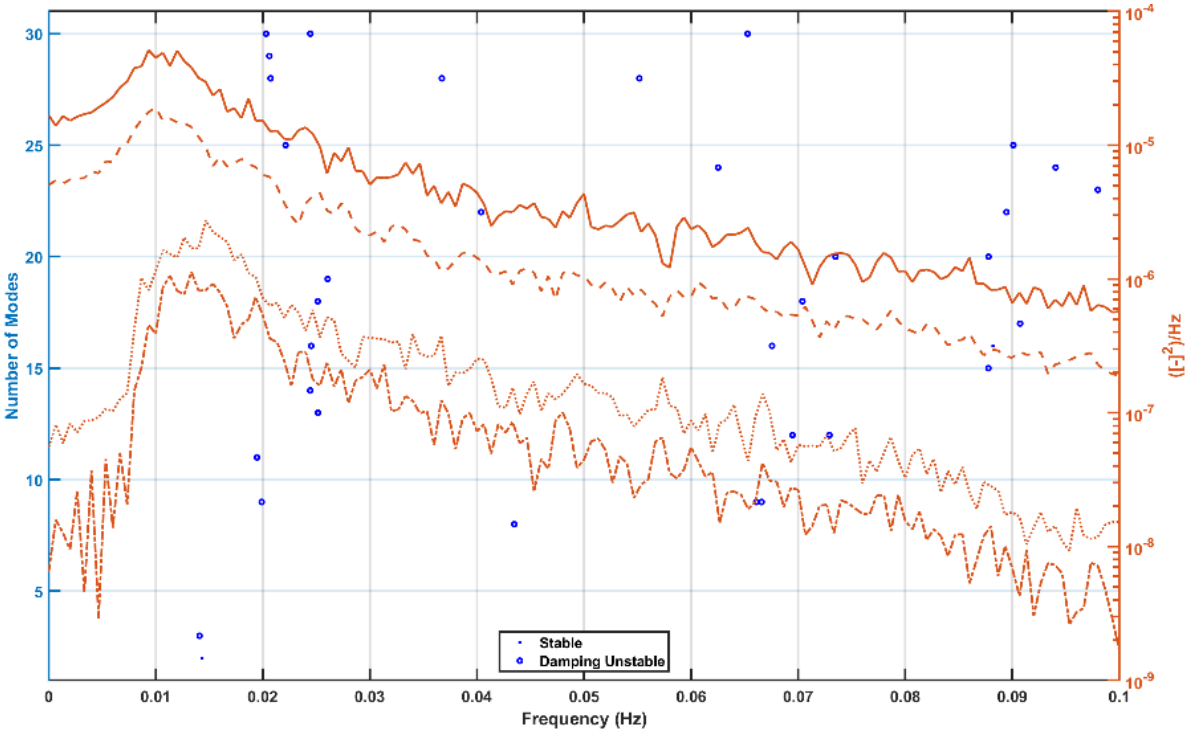

Number of Modes
Frequency (Hz)
([-]^2)/Hz
Stable
Damping Unstable

A1

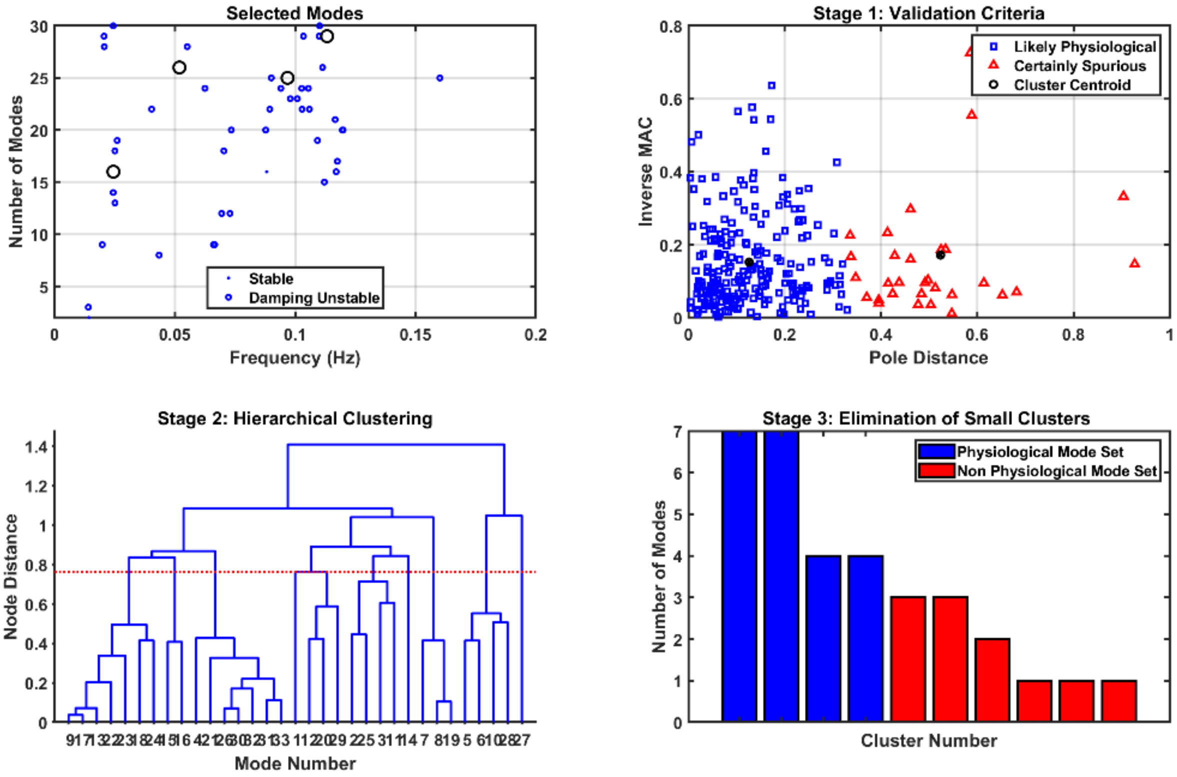

Selected Modes
Number of Modes
Frequency (Hz)
Stable
Damping Unstable
Stage 1: Validation Criteria
Inverse MAC
Pole Distance
Likely Physiological
Certainly Spurious
Cluster Centroid
Stage 2: Hierarchical Clustering
Node Distance
Mode Number
Stage 3: Elimination of Small Clusters
Number of Modes
Cluster Number
Physiological Mode Set
Non Physiological Mode Set

B1

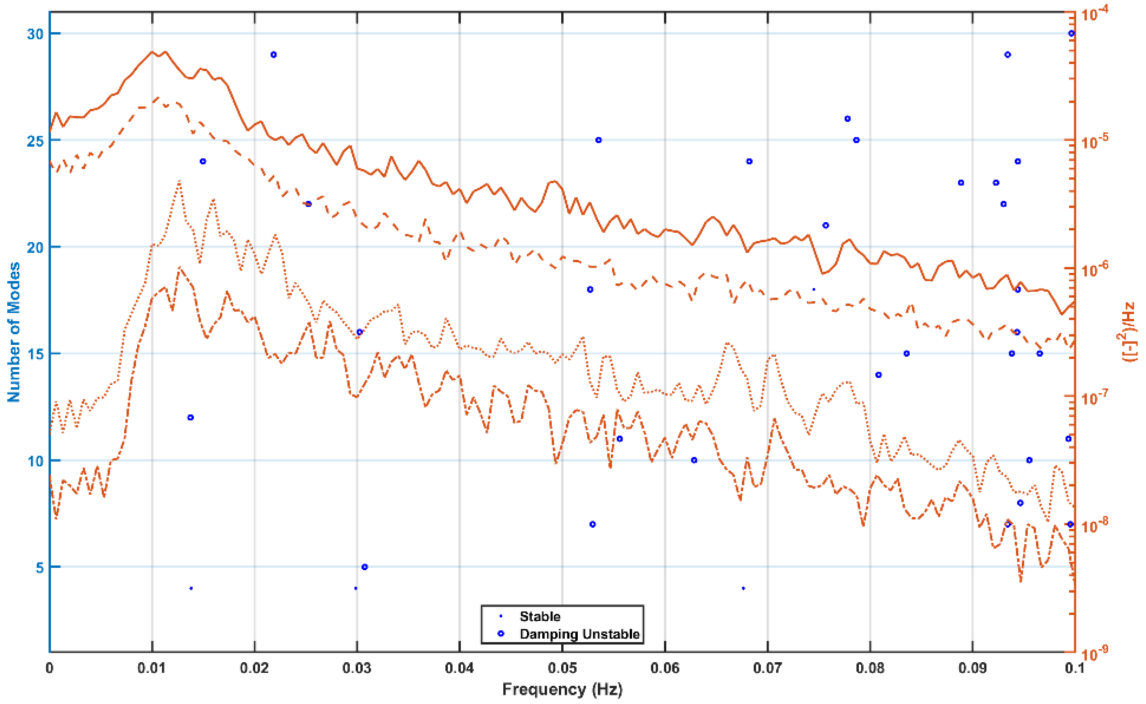
Number of Modes
Frequency (Hz)
([-]²)/Hz
Stable
Damping Unstable

A2

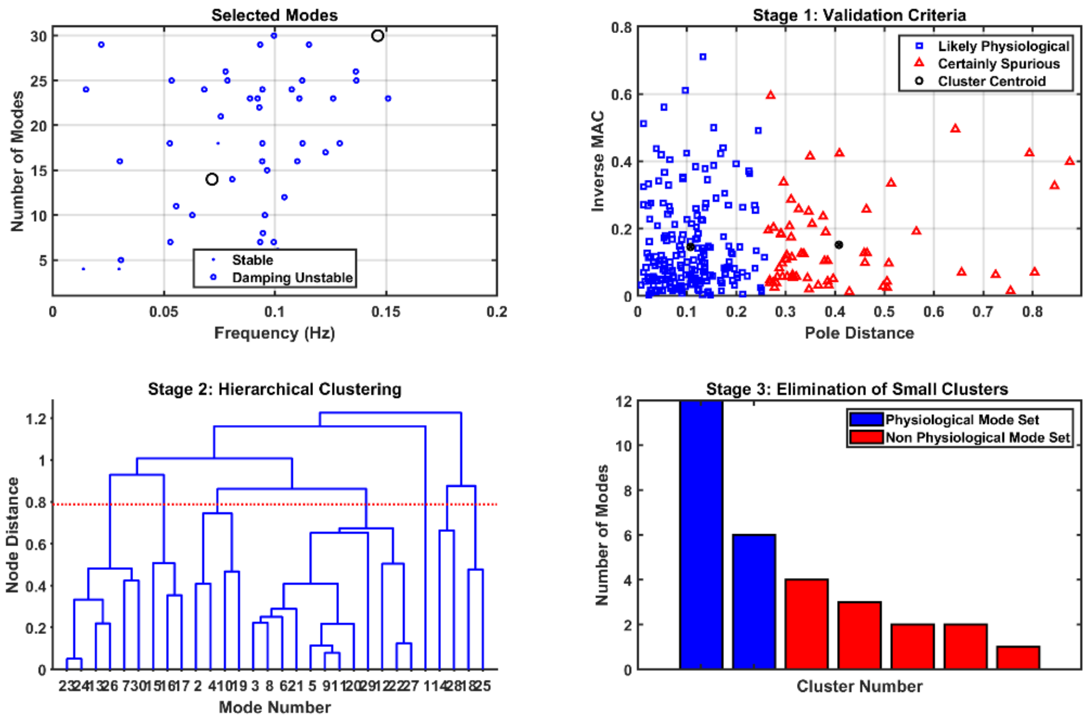
Selected Modes
Number of Modes
Frequency (Hz)
Stable
Damping Unstable
Stage 1: Validation Criteria
Inverse MAC
Pole Distance
Likely Physiological
Certainly Spurious
Cluster Centroid
Stage 2: Hierarchical Clustering
Node Distance
Mode Number
Stage 3: Elimination of Small Clusters
Number of Modes
Cluster Number
Physiological Mode Set
Non Physiological Mode Set

B2

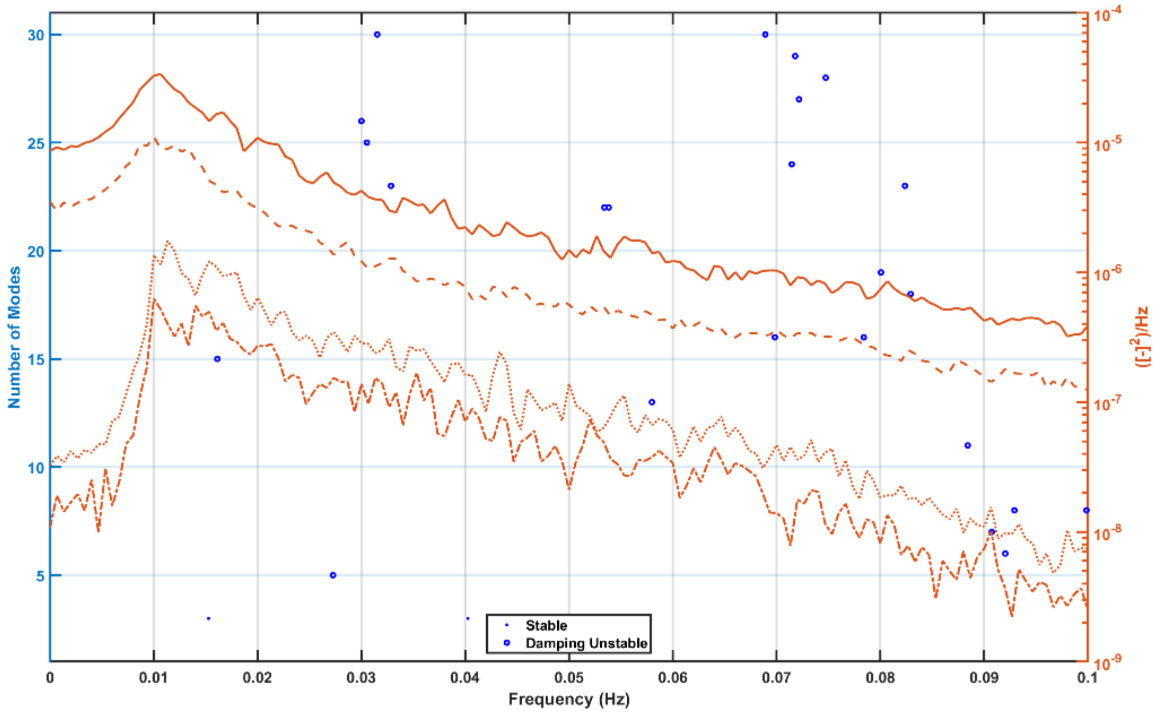
Number of Modes
([-]²)/Hz
Stable
Damping Unstable
Frequency (Hz)

A3

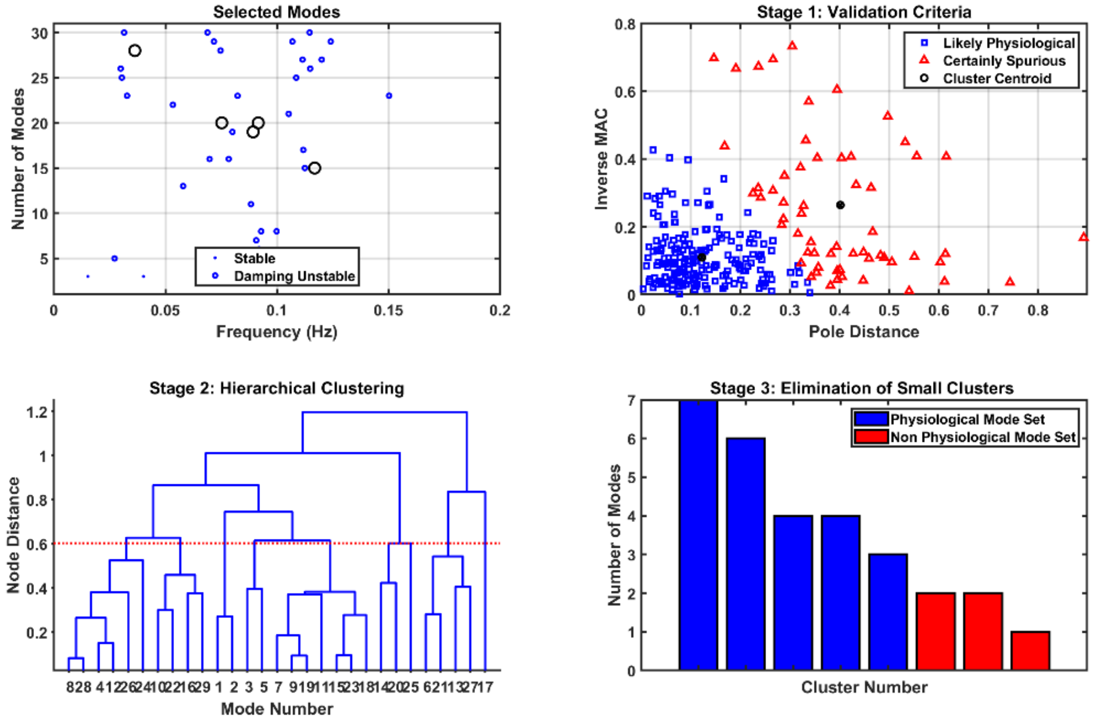
Selected Modes
Number of Modes
Stable
Damping Unstable
Frequency (Hz)
Stage 1: Validation Criteria
Likely Physiological
Certainly Spurious
Cluster Centroid
Inverse MAC
Pole Distance
Stage 2: Hierarchical Clustering
Node Distance
Mode Number
Stage 3: Elimination of Small Clusters
Physiological Mode Set
Non Physiological Mode Set
Number of Modes
Cluster Number

B3

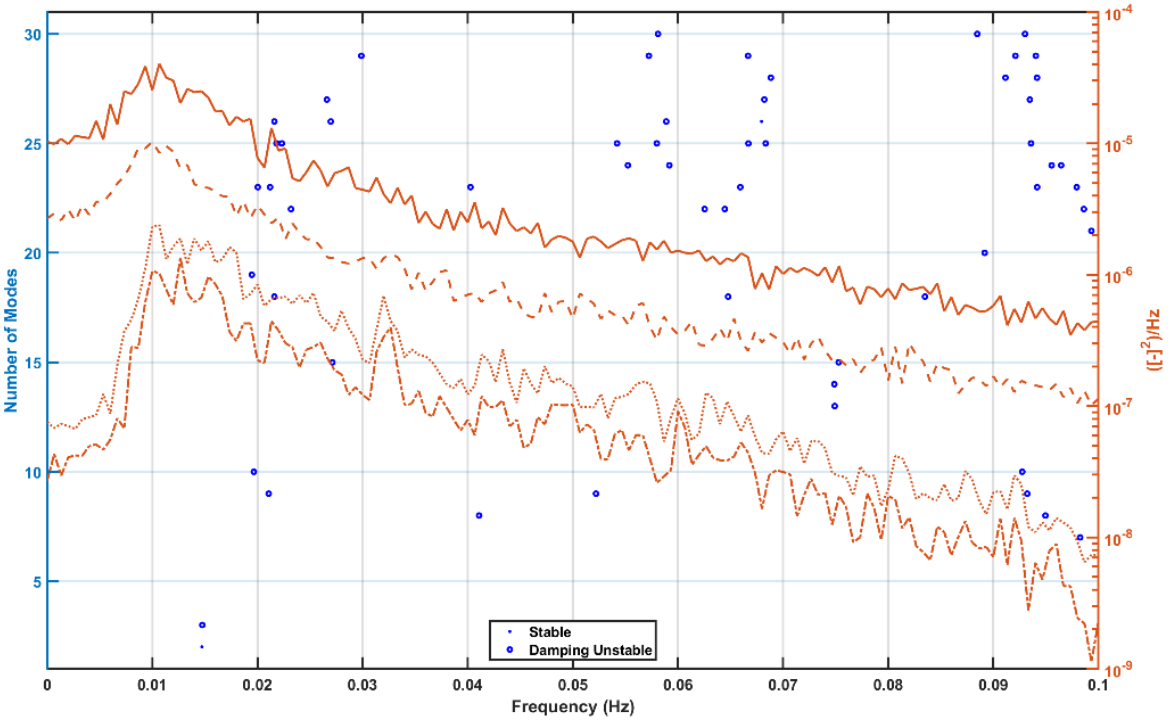

A4

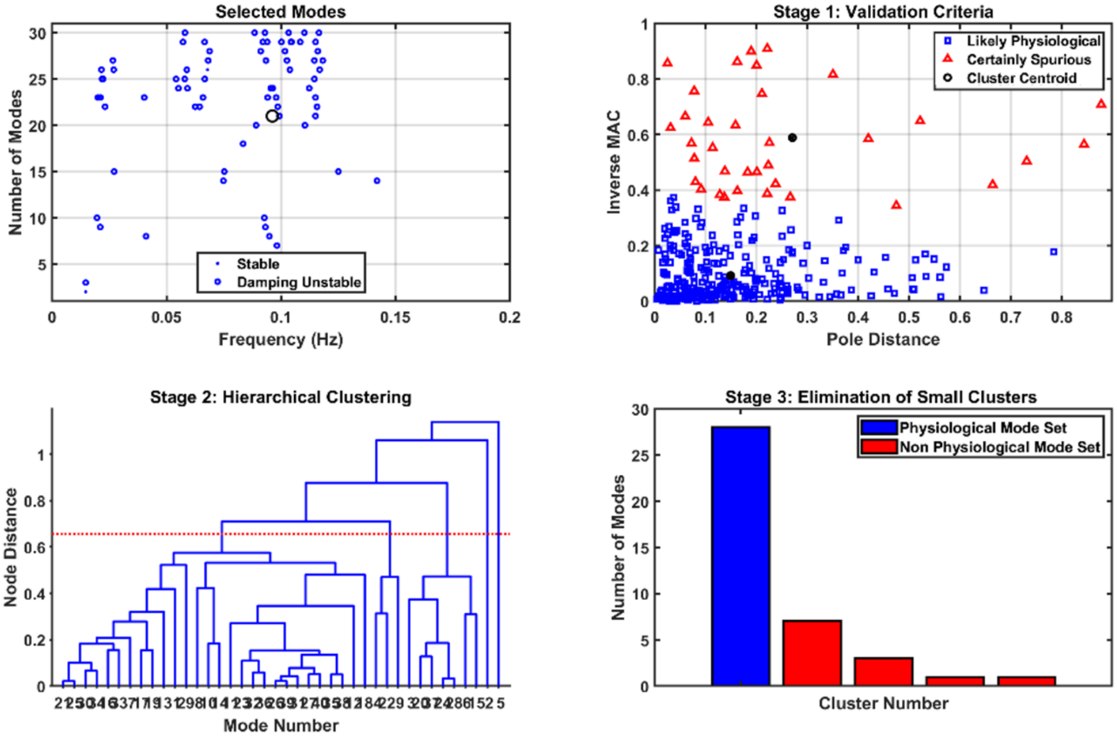

B4

Figure S2. Operational modal analysis (OMA) of near-infrared spectroscopy effects with maximum 30 modes for the power spectrum <0.1Hz in the stabilization diagram shown. Then, a multi-stage clustering for identifying physiological clusters using the Modal Toolkit (https://code.vt.edu/vibes-lab/modal-analysis). A1 and B1 are the stabilization diagram and the multi-stage clustering respectively for the Sedentary healthy control group. A2 and B2 are the stabilization diagram and the multi-stage clustering respectively for the Active

healthy control group. A3 and B3 are the stabilization diagram and the multi-stage clustering respectively for the T2DM intervention group pre-intervention. A4 and B4 are the stabilization diagram and the multi-stage clustering respectively for the T2DM intervention group post-intervention.

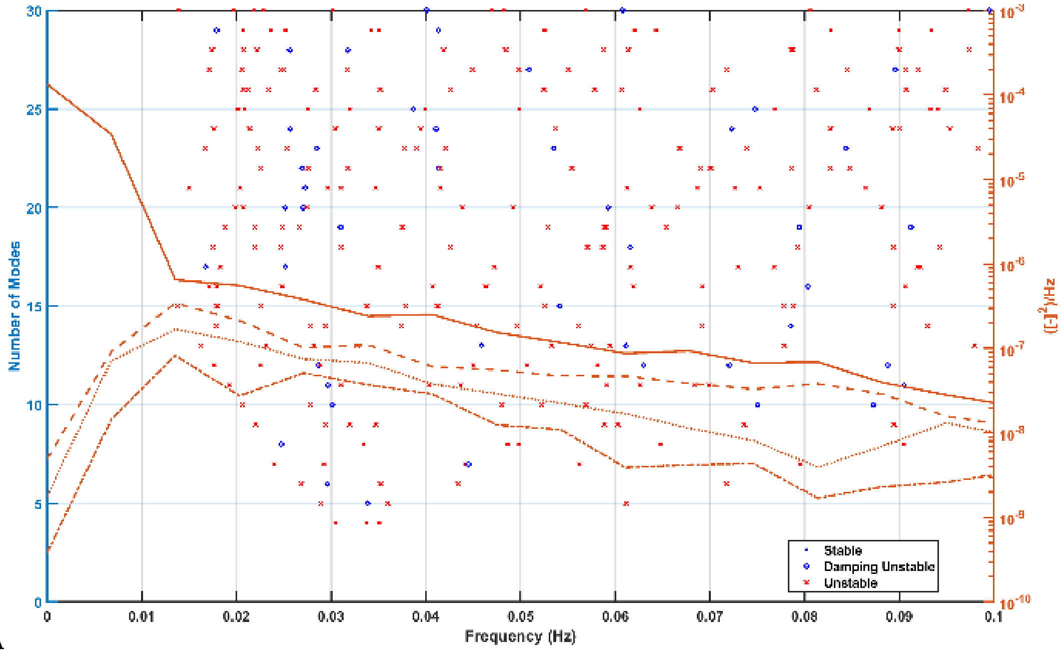

A

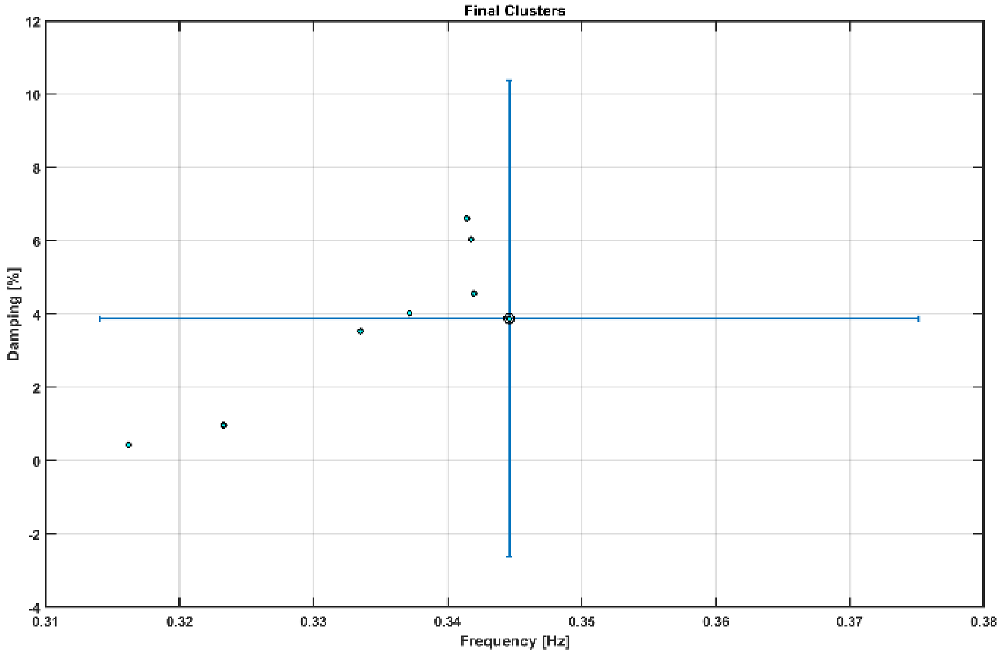

B

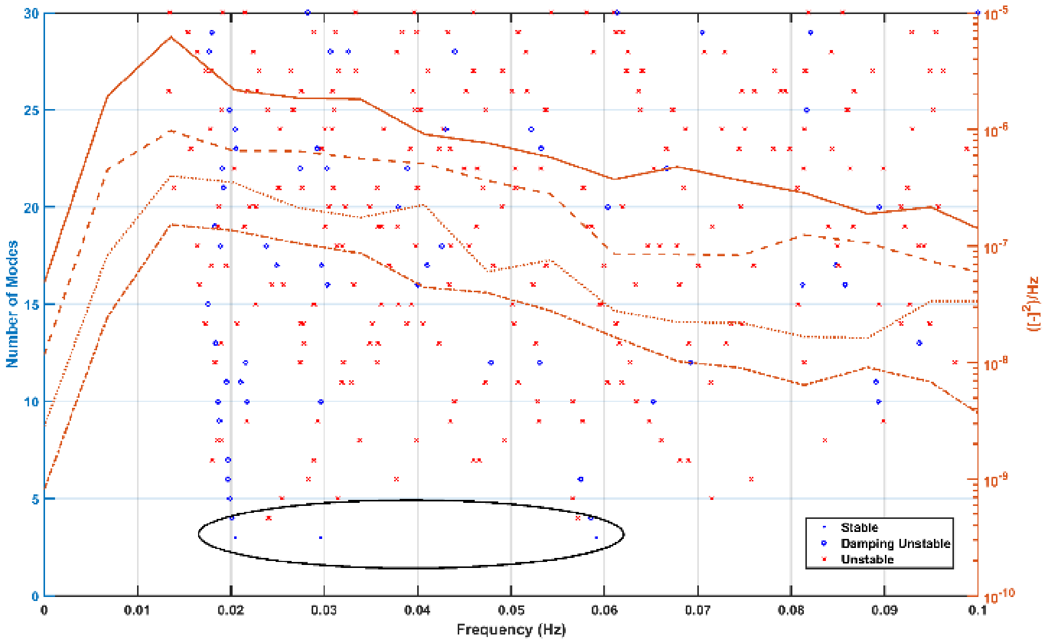

C

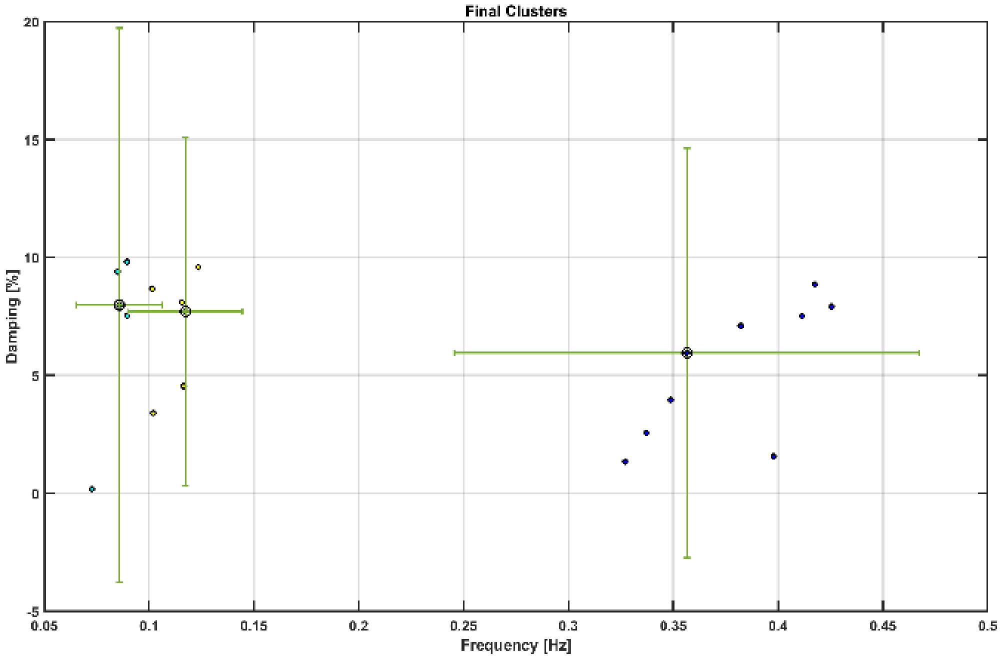

D

Figure S3. Operational modal analysis (OMA) of near-infrared spectroscopy signals from long-separation and short-separation channels with maximum 30 modes for the power spectrum <0.1Hz in the stabilization diagram shown for health control subjects (N=10). Then, a multi-stage clustering for identifying physiological clusters using the Modal Toolkit (https://code.vt.edu/vibes-lab/modal-analysis). A. stabilization diagram for the short-separation near-infrared spectroscopy channels, B. multi-stage clustering for the short-separation near-infrared spectroscopy channels. C. stabilization diagram for the long-

separation near-infrared spectroscopy channels, D. multi-stage clustering for the long-separation near-infrared spectroscopy channels.

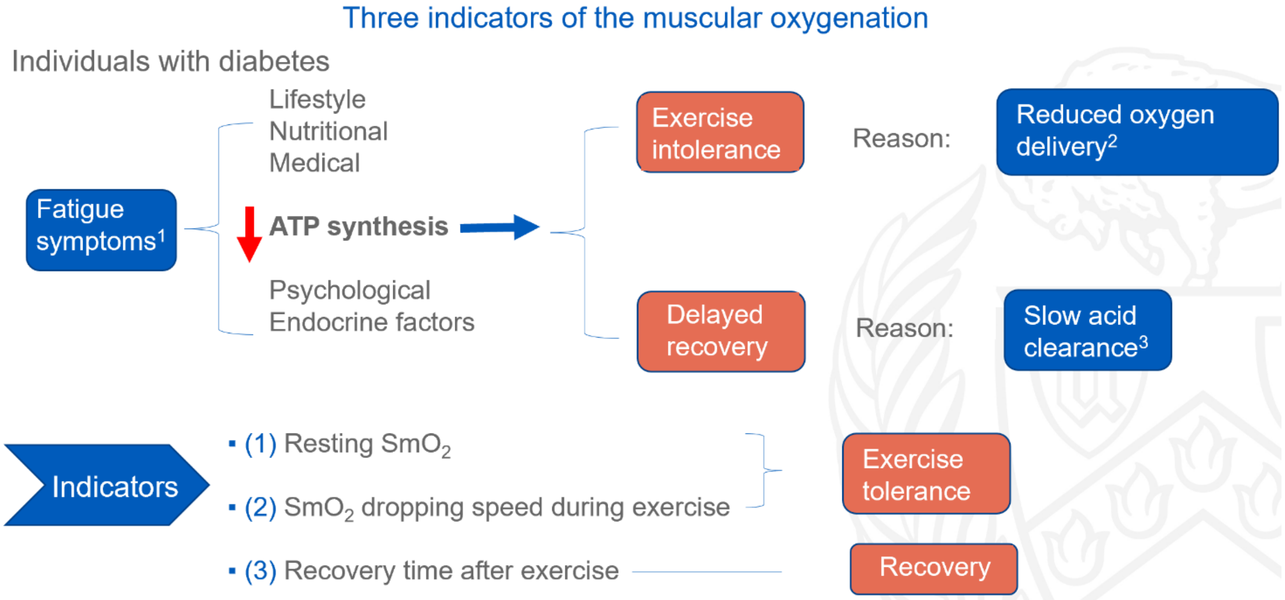

Figure S4. Postulated indicators of the muscle oxygenation response – resting SmO2, SmO2 drip during exercise, SmO2 recovery after exercise.

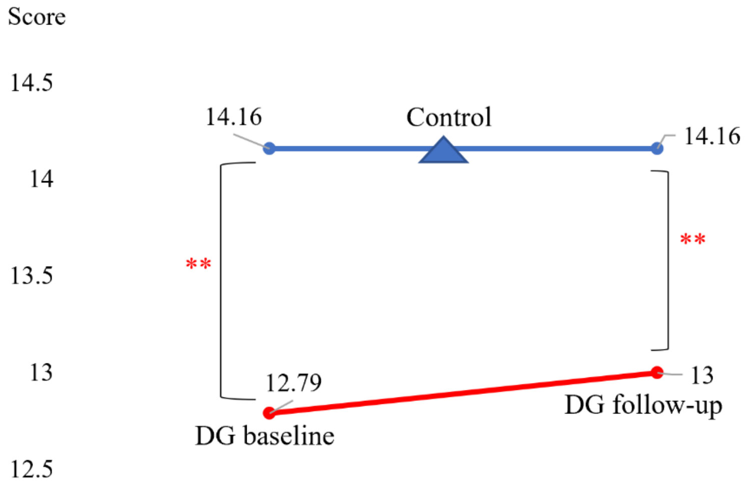

A.

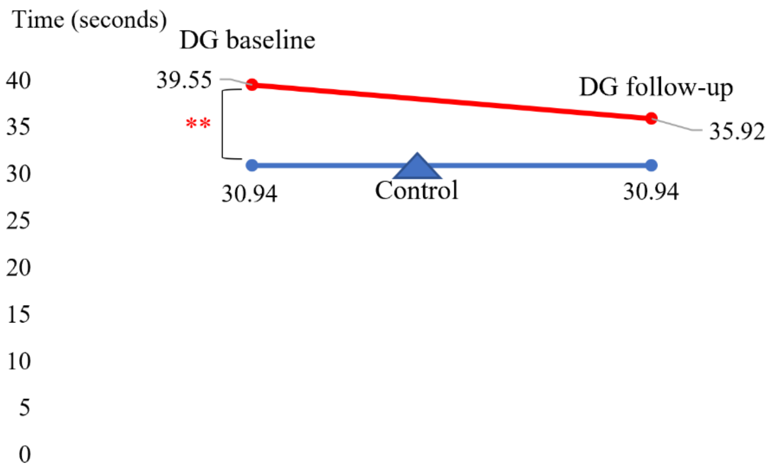

B.

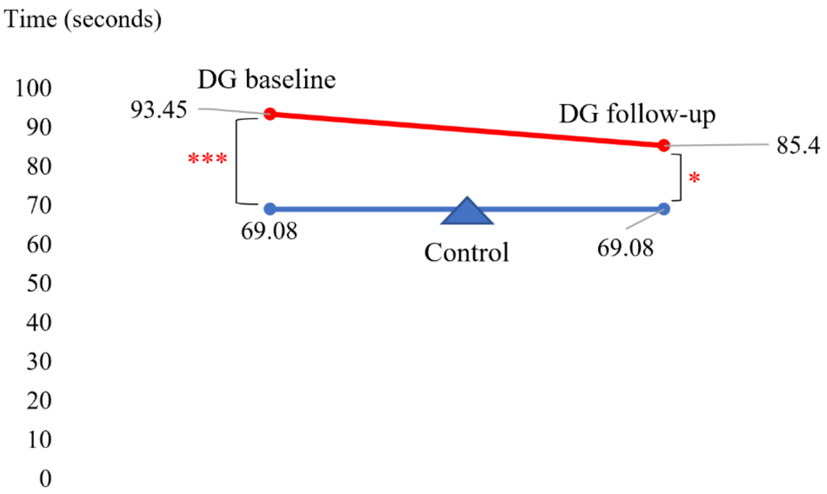

C.

Figure S5. Cognitive test comparisons between pre-intervention baseline and post-intervention. A. Mini-Cog. B. Trail making test Part A. C. Trail making test Part B. "DG" is the T2DM Intervention group, The sedentary healthy Control group and the active healthy are combined in the Control group. * $p < 0.05$, ** $p < 0.01$, *** $p < 0.001$.
